# Efficient and bright broadband electroluminescence based on environment-friendly metal halide nanoclusters

**DOI:** 10.1038/s41377-024-01427-z

**Published:** 2024-04-07

**Authors:** Dingshuo Zhang, Meiyi Zhu, Yifan He, Qingli Cao, Yun Gao, Hongjin Li, Guochao Lu, Qiaopeng Cui, Yongmiao Shen, Haiping He, Xingliang Dai, Zhizhen Ye

**Affiliations:** 1 School of Materials Science and Engineering, State Key Laboratory of Silicon and Advanced Semiconductor Materials, Zhejiang University, 310027 Hangzhou, China; 2 Wenzhou Key Laboratory of Novel Optoelectronic and Nano Materials and Engineering Research Centre of Zhejiang Province, Institute of Wenzhou, Zhejiang University, 325006 Wenzhou, China; 3 Wenzhou XINXINTAIJING Tech. Co. Ltd., 325006 Wenzhou, China; 4 Department of Chemistry, Zhejiang Sci-Tech University, 310018 Hangzhou, China

**Keywords:** Lasers, LEDs and light sources, Optical materials and structures

## Abstract

Broadband electroluminescence based on environment-friendly emitters is promising for healthy lighting yet remains an unprecedented challenge to progress. The copper halide-based emitters are competitive candidates for broadband emission, but their high-performance electroluminescence shows inadequate broad emission bandwidth of less than 90 nm. Here, we demonstrate efficient ultra-broadband electroluminescence from a copper halide (CuI) nanocluster single emitter prepared by a one-step solution synthesis-deposition process, through dedicated design of ligands and subtle selection of solvents. The CuI nanocluster exhibits high rigidity in the excitation state as well as dual-emissive modes of phosphorescence and temperature-activated delayed fluorescence, enabling the uniform cluster-composed film to show excellent stability and high photoluminescent efficiency. In consequence, ultra-broadband light-emitting diodes (LEDs) present nearly identical performance in an inert or air atmosphere without encapsulation and outstanding high-temperature operation performance, reaching an emission full width at half maximum (FWHM) of ~120 nm, a peak external quantum efficiency of 13%, a record maximum luminance of ~50,000 cd m^−2^, and an operating half-lifetime of 137 h at 100 cd m^−2^. The results highlight the potential of copper halide nanoclusters for next-generation healthy lighting.

## Introduction

Electroluminescent devices with ultrabroad emission are considered promising candidates for the next-generation healthy lighting technology^1^. Traditional solid-state lighting sources utilize photoluminescence down conversion technology to realize ideal white emission that simultaneously possesses a high color rendering index (CRI), high luminous efficiency of radiation (LER), and appropriate correlated color temperature (CCT)^2,3^. In this technology, yellow phosphors are excited by blue light-emitting diodes (LEDs), or three-primary-color phosphors are excited by ultraviolet LEDs, suffering the instability of emission spectra that may shift the CRI and CCT due to the discrepant degradation rates of the emitters^4^. Rare-earth metals (e.g., Y, Ce)^5,6^ or toxic metals (e.g., Cd, Pb)^7,8^ are always used in the phosphors. Moreover, the intense blue-violet components in their emission are harmful to healthy lighting^9,10^. Therefore, developing electroluminescent devices with ultrabroad and spectra-stable emission based on environment-friendly emitters is of importance for future healthy lighting. Over the past few years, several kinds of double perovskites (e.g., Cs_2_AgInCl_6_)^11,12^, heterophase α/δ-CsPbI_3_ (ref. ^13^.), and perovskite derivant (e.g., CsCu_2_I_3_)^14,15^ were discovered to present broadband emissions originating from self-trapped excitons. However, their electroluminescent performance is far inferior to lighting applications^15–17^, which is considered a result of low carrier mobility and wide bandgap of the emitters. To date, realizing efficient and spectra-stable ultra-broadband LEDs remains an unprecedented challenge^18^.

Ligand-stabilized nanoclusters combine the stability of inorganic particles and the diversity of organic ligands to exhibit good tunability of luminescence spectra and superiority in rigidity and photostability, showing great potential in achieving ultra-broadband LEDs^19–22^. Specifically, copper iodide (CuI) nanocluster-based LEDs present a broad EL spectrum with a full width at half maximum (FWHM) up to 180 nm (ref. ^23^.). Nevertheless, the device showed an inferior external quantum efficiency (EQE) of 0.73%. Highly efficient nanocluster-based LEDs can be achieved in devices exhibiting a narrow FWHM below 90 nm (refs. ^24–26^.). Furthermore, the fabrication of the LEDs based on CuI nanoclusters requires thermal evaporation or solution deposition, which calls for excellent thermal stability or solvent solubility of the nanoclusters. Unfortunately, most of the nanoclusters show inferior stability under thermal evaporation temperatures^20^, and the solubility of nanoclusters is largely limited by the ligands and their rigid structure, leading to poor processability^23,27^. Specific synthesis and purification processes are required for the preparation of CuI nanoclusters.

Here, we develop a strategy of synthesis in one-step solution deposition of CuI nanoclusters for fabricating high-performance ultra-broadband LEDs. Through the dedicated design of ligands and selection of solvents, bright and uniform films consisting of CuI nanoclusters and ligand hosts are achieved, showing excellent thermal stability and atmosphere-resistant characteristics. The resulting LEDs present nearly identical performance in the nitrogen-filled and air atmosphere without encapsulation, exhibiting an FWHM of ~120 nm, a peak EQE of 13%, a maximum luminance of nearly 50,000 cd m^−2^, and an operating half-life of up to 137 h at 100 cd m^−2^.

## Results

### Synthesis in one-step solution deposition of CuI nanoclusters

We recognize that inorganic CuI can be feasibly coordinated with chelate pyridine or phosphine ligands^28–30^, and the CuI and a few ligands are soluble in organic solvents. It is likely to synthesize the CuI nanoclusters during solution-processed film deposition by controlling the reaction between CuI and ligands, thus avoiding specific synthesis and thermal evaporation processes of the CuI nanoclusters. Key to in-situ fabricating the nanocluster-composed film is the dedicated design of ligands and subtle selection of solvents, which guarantee the initiation of the reaction to generate the nanocluster during film deposition rather than in the precursor solution.

**Fig. 1:**
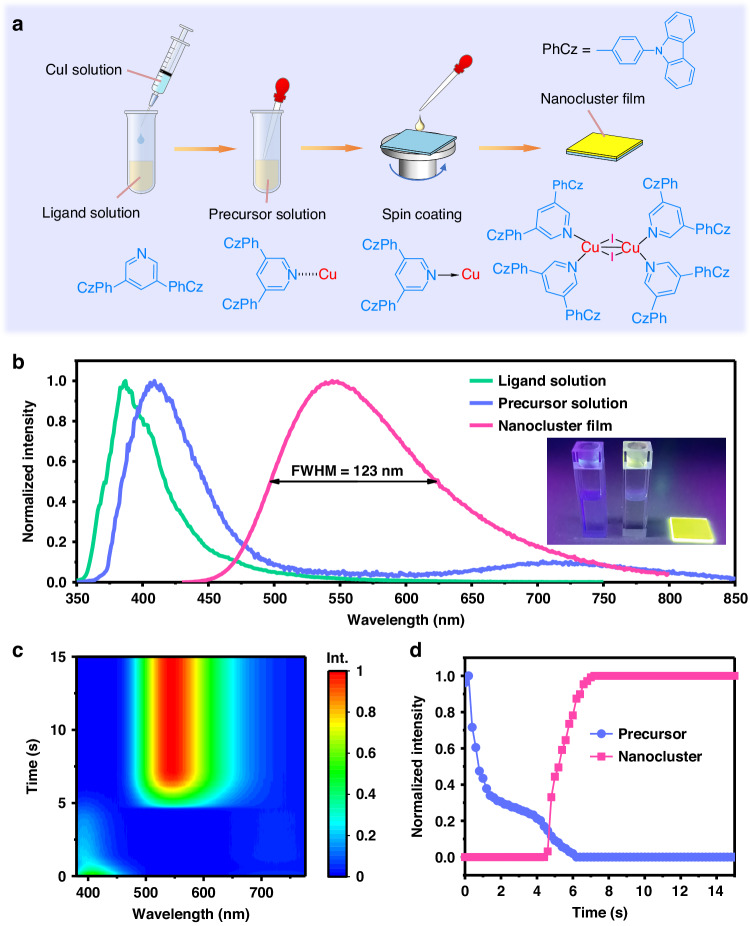
One-step synthesis-deposition process. **a** Schematic diagram of the one-step synthesis-deposition process to produce 35DCzPPy-chelated CuI nanocluster films. **b** Normalized steady-state PL spectra of the ligand solution, precursor solution, and the as-prepared film under an excitation wavelength of 260 nm. The inset shows the corresponding photographs in order from left to right. **c** Evolution of the PL spectra during the spin-coating process under the excitation wavelength of 310 nm. **d** Evolution of normalized PL intensity of the deposited precursor solution and the nanoclusters extracted from the spectra in c

Figure 1a displays the detailed procedures of the one-step synthesis-deposition process. The CuI nanocluster-composed film is fabricated by directly mixing the CuI solution and the ligand solution to obtain the precursor solution, followed by spin-coating the precursor solution on the substrates. The bright film can be feasibly obtained without further thermal annealing (Fig. 1b). Since the nanocluster-composed film is targeted for use as an emissive layer in an LED, the carrier transport of the nanocluster-composed film is also considered. Therefore, a bipolar conductive molecule that contains pyridine anchoring groups, namely 3,5-*bis*(3-(9H-carbazol-9-yl)phenyl)pyridine (35DCzPPy), is designed as the candidate ligand for the CuI nanocluster (Supplementary Fig. S1). Compared with similar 3,5-*bis*(carbazol-9-yl)pyridine (mCPy) ligand reported in literature^29^, the 35DCzPPy possess an additional benzene ring between the carbazole and pyridine, enabling enhanced conjugated effect and thus the improved conductivity. It is known that the 35DCzPPy is an excellent host material for organic LEDs^31,32^. We expect the formation of a dopant-host structured film, in which the in-situ generated CuI nanoclusters play the dopant and the excess uncoordinated ligands play the host. Regarding the choice of solvent, chlorobenzene and acetonitrile are selected to dissolve 35DCzPPy and CuI, respectively, after careful screening of the solubility of 35DCzPPy and CuI as well as the volatility of the solvents (Supplementary Tab. S1). We find that chlorobenzene can dissolve the 35DCzPPy but hardly dissolve the CuI, and vice versa for the acetonitrile. After mixing the CuI-acetonitrile solution and the 35DCzPPy-chlorobenzene solution, the precursor solution presents a red-shifted photoluminescence (PL) spectrum compared with the original ligand solution but without the formation of nanoclusters (Fig. 1b). The PL red-shift is presumed to originate from the interaction between the ligand and CuI in the solution and the weak PL component observed at 600–850 nm is ascribed to the triplet cluster-centered (^3^CC) excited state of incompletely coordinated Cu clusters^33,34^. In contrast, the nanoclusters directly form and precipitate if using dimethyl sulfoxide or N, N-dimethylformamide that dissolves both CuI and 35DCzPPy as the solvent (Supplementary Fig. S2), which cannot enable the formation of the nanocluster-composed film. On the other hand, volatile solvents (e.g., tetrahydrofuran and dichloromethane), which can also dissolve the 35DCzPPy but hardly dissolve the CuI, bring difficulty in film thickness and quality control. The optimized volume ratio of the CuI-acetonitrile solution and the 35DCzPPy-chlorobenzene solution is 1 to 4, as the precursor solution is stable and moderately volatile to facilitate film deposition.

We highlight the critical importance of the type of ligands in forming the bright CuI nanocluster. The 35DCzPPy-anchored nanoclusters exhibit broadband emission at 550 nm with an FWHM up to 123 nm (Fig. 1b), showing the highest PL quantum yield (PL QY) of 60% for the film. The host-guest luminescence mechanism is evidenced by the distinct reduction of the PL lifetime of 35DCzPPy in a pure 35DCzPPy film compared with a nanocluster film prepared by the in-situ synthesis-deposition method (Supplementary Fig. S3). Another three representative molecules, 2,6-*bis*(3-(9H-carbazol-9-yl)phenyl)pyridine (26DCzPPy), 1,3,5-*tri*(m-pyridine-3-ylphenyl)benzene (TmPyPB) and 2,9-di(diphenylphosphine)-dibenzofuran (DBFDP) are selected as comparative ligands (Supplementary Fig. S1), where the former two contain pyridine anchoring group and the latter is a phosphine-based ligand. PL spectra of these ligands, the corresponding precursor solutions, and nanocluster films are shown in Supplementary Fig. S4. Due to a large steric hindrance near the N atom of the pyridine group, 26DCzPPy cannot effectively coordinate with CuI to form a bright nanocluster film, although it shares a similar molecular structure with 35DCzPPy. In the case of TmPyPB, its unipolar conductivity makes it inappropriate as a host to effectively confine excitons, and instead acts as a role of emission quencher, resulting in nanocluster films with short exciton lifetime and low emission efficiency (Supplementary Fig. S3). For the diphosphine chelate DBFDP, its undesirable molecular design cannot effectively suppress inefficient emission from the triplet cluster-centered excited state and thus limits the PL QY of nanoclusters^22^. Notably, the PL spectra and PL QYs of the nanocluster-composed films can also be modulated by the molar ratio of ligand to CuI in the precursor solution (Supplementary Fig. S5). The optimum molar ratio of ligand to CuI is 2.7:1, which serves as a basis for the following discussions.

We then investigate the formation process of CuI nanocluster through in-situ monitoring of PL spectra of the precursor during film deposition. An ultraviolet LED (310 nm) is used to excite the samples and a spectrometer coupled with fiber records the PL spectra and intensity with a time resolution of 0.1 s (Supplementary Fig. S6). During the first 4.0 s of the spin-coating step, the precursor solution is rapidly spun away from the substrates, resulting in a rapid reduction in PL strength (Fig. 1c and Supplementary Fig. S6). No nanocluster emission is detected at this stage, at which viscous force, instead of solvent evaporation, governs the thinning of the wet precursor film. From 4.6 s after the beginning of spin-coating, the PL peak centered around 550 nm starts to occur and intensifies rapidly (Fig. 1d), identifying the formation of the nanoclusters. After 7.2 s, the precursor emission disappears and the nanocluster emission saturates, suggesting the full conversion from precursor to nanoclusters. The short conversion time indicates the readily reaction between the CuI and 35DCzPPy, consistent with the strong interaction between the Cu atoms and pyridine groups. We note that the reaction is initiated by supersaturation of the precursor induced by rapid volatilization of the solvent, which reduces the intermolecular distance. This aggregate-induced reaction is confirmed by the formation of the nanocluster during the evaporation of the precursor solution under heating (Supplementary Fig. S7).

### Uniform and compact CuI nanocluster-composed film

**Fig. 2:**
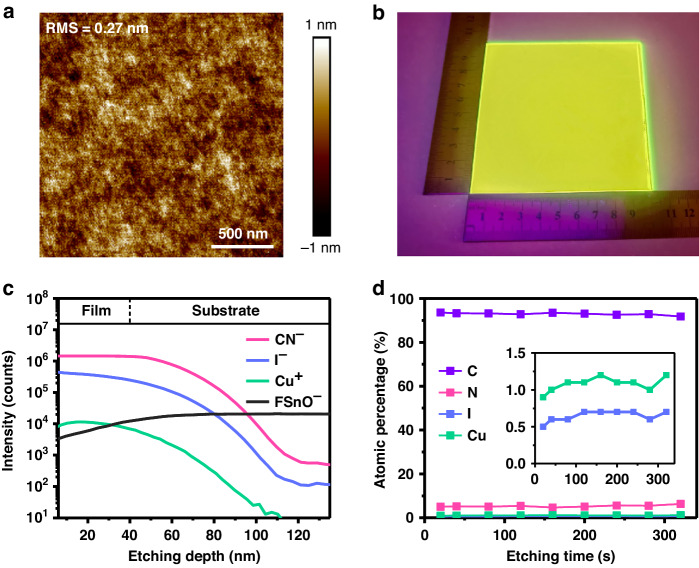
Uniform and smooth CuI nanocluster films. **a** AFM image. **b** Digital photo of a film with an area of 10 × 10 cm^2^ deposited by blade coating in the air atmosphere. The excitation wavelength is 254 nm. **c** TOF-SIMS depth profile of the film deposited on the fluorine-doped tin oxide (FTO) substrate. **d** Atomic percentages of the four constituent elements C, N, I, and Cu at different etching times. The inset is to show the less abundant I and Cu elements more clearly. Considering the surface contamination of the film, the data are collected from the 20 seconds after etching

The film morphology is characterized by atomic force microscopy (AFM) and scanning electron microscopy (SEM). AFM image (Fig. 2a) shows that the film is highly smooth with a root-mean-square roughness as low as 0.27 nm, which is comparable to that of the films prepared by vacuum thermal evaporation. As shown in SEM images (Supplementary Fig. S8), the film is dense and continuous without pinholes. These results suggest excellent film-forming properties using the mixed chlorobenzene and acetonitrile solvents, motivating us to fabricate large-area films. A film with an area of 10 × 10 cm2 deposited by blade coating (see Methods for details) also shows bright and uniform luminance (Fig. 2b), indicating the universality of synthesis in one-step solution deposition of CuI nanoclusters.

To reveal the homogeneity of the film from a vertical point of view, time-of-flight secondary ion mass spectrometer (TOF-SIMS) characterizations and depth profiling X-ray photoelectron spectroscopy (XPS) measurements were carried out. Demonstrated by the TOF-SIMS depth profile, the distribution of N, I, and Cu are found to be fairly uniform throughout the thin film (Fig. 2c). The depth-dependent XPS curves show that the atomic percentage of all the constituent elements remains almost constant until the underneath film (Fig. 2d and Supplementary Fig. S9), indicating that the nanoclusters are evenly distributed in the film without segregation. The results above prove that the in-situ deposited nanocluster films possess outstanding homogeneity.

### Excited-state characteristics and photophysical properties

**Fig. 3:**
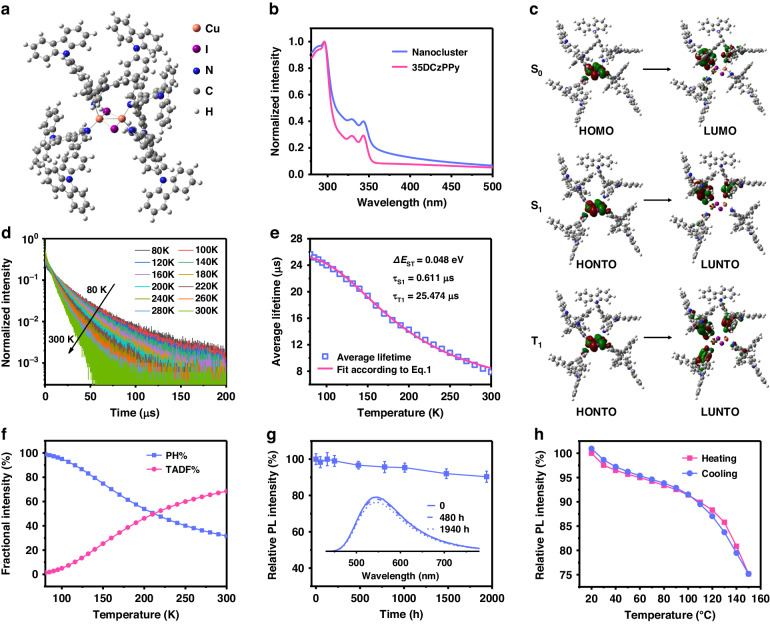
Excited-state characteristics and photophysical properties of [35DCzPPy]_4_Cu_2_I_2_. **a** Optimized structure of [35DCzPPy]_4_Cu_2_I_2_ with a size around 2.3–2.6 nm. **b** Normalized UV−vis absorption spectra of nanocluster and 35DCzPPy films. **c** DFT and TDDFT analysis of the ground state (S_0_) characteristics and the singlet and triplet transitions of [35DCzPPy]_4_Cu_2_I_2_. HONTO and LUNTO are the highest occupied and lowest unoccupied natural transition orbital (NTO), respectively. S_0_, S_1_, and T_1_ refer to the ground state and the first singlet and triplet excited states. **d** Normalized time-resolved PL decay curves of [35DCzPPy]_4_Cu_2_I_2_. **e** Average lifetime variation in the temperature ranges from 80 to 300 K, and a fit of the data according to Eq. (1). The calculated parameters are shown in the figure. **f** Temperature-dependent fractional contributions of PH and TADF to the emission of [35DCzPPy]_4_Cu_2_I_2_. **g** Evolution of PL intensity of the unencapsulated film stored in the air at different times. **h** Evolution of PL intensity during heating and then cooling in the range of 20 to 150 °C in a vacuum. To ensure that the temperature of the sample is consistent with the indicated one, each temperature is held for 5 min before testing

The molecular structure of CuI nanoclusters is shown in Fig. 3a based on the previous clusters with analogous ligands^29^ and optimized by density functional theory (DFT) simulation, which is further verified by extended X-ray absorption fine structure (EXAFS; Supplementary Fig. S10 and Tab. S2; see Methods for details of sample preparation and data analysis). The nanocluster is denoted as [35DCzPPy]_4_Cu_2_I_2_, with Cu_2_I_2_ as the core and two ligands chelated to each Cu atom. The chelation between CuI and 35DCzPPy is evidenced by the XPS core-level spectrum (Supplementary Fig. S11). Time-dependent DFT (TDDFT) simulations on the first singlet (S_1_) and triplet (T_1_) excited states of [35DCzPPy]_4_Cu_2_I_2_ are performed to figure out its configuration variation (Supplementary Tab. S3). Compared with the ground state, the geometry of Cu_2_I_2_ under the excited states is almost constant, showing high rigidity of the nanocluster and the corresponding effective inhibition of excited-state energy loss caused by structural relaxation. The absorption spectra of the nanocluster and 35DCzPPy films (Fig. 3b) show identical peak positions, indicating that the absorption of the nanocluster is dominated by ligands. A distinct tail from 350 to 400 nm can be observed from the spectrum of the nanocluster film, which is attributed to metal-to-ligand charge transfer (MLCT) transitions^35,36^. The frontier molecular orbital (FMO) levels of [35DCzPPy]_4_Cu_2_I_2_ are experimentally evaluated as −5.8 and −2.3 eV for the highest occupied and the lowest unoccupied molecular orbitals (HOMO and LUMO), respectively, through ultraviolet photoelectron spectroscopy (Supplementary Fig. S12). FMO analysis of the ground state (S_0_) and natural transition orbital (NTO) analysis of S_1_ and T_1_ states are carried out (Fig. 3c). HOMO and LUMO of the nanocluster are separated, located on the Cu_2_I_2_ core and pyridine groups of two ligands with overlapping on the coordinated Cu and N atoms, suggesting that metal-and-counterion-to-ligand charge transfer ((M + X)LCT) components are predominant in the lowest excited states. For the S_0_ → S_1_ and S_0_ → T_1_ excitations, the spatial distribution of the highest occupied NTOs (HONTO) remains almost constant compared with HOMO at the ground state, whereas the lowest unoccupied NTOs (LUNTO) expand to the benzene ring and further to all the four ligands at S_1_ and T_1_ states, respectively. Therefore, it can be expected that the enhanced overlap integral (<Ψ_H_|Ψ_L_>) for the S_0_ → T_1_ excitation could increase the probability of phosphorescence (PH) radiation.

We conduct temperature-dependent time-resolved photoluminescence characterizations to reveal the luminescent mechanism of the nanocluster. The emission lifetime (*τ*) shows a strong temperature dependence, which decreases sharply from 25.7 μs at 80 K to 7.9 μs at 300 K (Fig. 3d). This result suggests an intense enhancement of the thermally activated population of the S_1_ state. To determine the singlet-triplet splitting and the individual emission decay times of the nanocluster, the variation of lifetimes with respect to temperature can be fitted according to the following equation^37^:

1 $$\tau \left(T\right)=\frac{3+\exp \left(-\frac{\varDelta {E}_{\text{ST}}}{{k}_{\text{B}}T}\right)}{\frac{3}{\tau \left({\text{T}}_{1}\right)}+\frac{1}{\tau \left({\text{S}}_{1}\right)}\exp \left(-\frac{\varDelta {E}_{\text{ST}}}{{k}_{\text{B}}T}\right)}$$

with *ΔE*_ST_ the singlet-triplet splitting energy. As shown in Fig. 3e, the well-matching fit curve reveals the individual emission decay times and energy separations between the singlet and triplet states. *ΔE*_ST_ as low as 0.048 eV enables efficient thermally activated delayed fluorescence (TADF) by facilitating reverse intersystem crossing. The contributions of PH and TADF to the PL of the nanocluster are calculated according to the fitted data and shown in Fig. 3f (see detailed calculations in Methods). The nanocluster shows dual-mode radiation with PH and TADF accounting for 32% and 68% at room temperature, respectively. It is believed that dual emissive dyes, i.e., emitters that simultaneously harvest singlet and triplet excitons to generate both PH and TADF have greater potential in inhibiting exciton accumulation and quenching than mono emissive dyes^38,39^. Hence, the nanocluster possesses great potential in realizing LEDs with a low efficiency roll-off, high brightness, and good stability due to the suppression of exciton quenching. Notably, the presence of numerous charge transfer processes with different energy levels results in a broad energy distribution for the ultimate radiation, leading to the efficient and broadband emission.

Besides, the ambient resistance and thermal stability of the [35DCzPPy]_4_Cu_2_I_2_ nanocluster-composed film were investigated. Cu^+^ is usually considered unstable in ambient air, and prone to facile conversion into Cu^2+^. Notably, after being stored in the air for three months, the PL intensity of the film maintains 90% of the original value, and the PL spectra remain identical, indicating the great chemical stability of the nanocluster (Fig. 3g and Supplementary Fig. S13). Regarding thermal stability, the PL intensity of the film can be completely recovered when suffering a high temperature of 150 °C (Fig. 3h). Remarkably, the PL intensity at 100 °C maintains above 90% of the original value at room temperature, indicating the excellent thermal resistance of the nanocluster. These results point to the CuI nanocluster as an outstanding phosphor possessing broadband emission, air-stable, and thermal-stable characteristics.

### Efficient and stable ultra-broadband LEDs

**Fig. 4:**
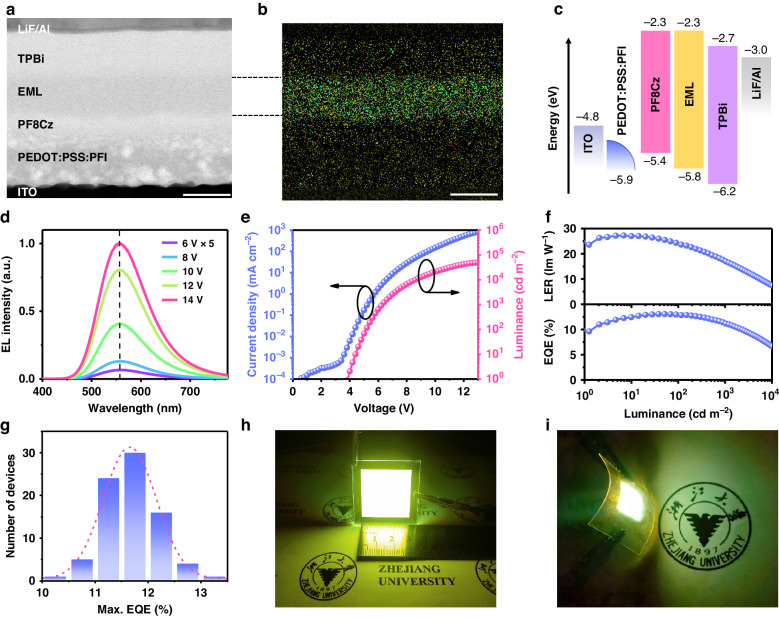
LED performance. **a**, **b** High-angle annular dark-field (HAADF) image (**a**) and the corresponding EDX image (**b**) (Cu and I are denoted as yellow and green, respectively) of a cross-sectional sample of the LED. Scale bar: 50 nm. **c** Flat-band energy level diagram. **d** EL spectra under forward biases of 6, 8, 10, 12, and 14 V. **e**, **f** Current density–luminance–voltage characteristics (**e**) and the corresponding LER/EQE–luminance relationship (**f**) of the champion device. **g** Histogram of peak EQEs measured from 81 devices from 9 batches. **h**, **i** Digital photos of a large-area device with an active size of 3 × 3 cm^2^ (**h**) and a flexible device fabricated on an ITO–PET substrate (**i**)

EL properties of the nanoclusters are verified based on LEDs with a multilayered structure (Fig. 4a–c) of indium tin oxide (ITO, ~60 nm)/poly(3,4-ethylenedioxythiophene): poly(styrene sulfonate): perfluorinated resin (PEDOT:PSS:PFI, ~60 nm)/poly((9,9-dioctylfluorenyl-2,7-diyl)-alt-(9-(2-ethylhexyl)-carbazole-3,6-diyl)) (PF8Cz; crosslinked, ~15 nm)/nanocluster-composed film ( ~ 40 nm)/2,2′,2″(l,3,5-benzenetriyl)-tris(L-phenyl-l-H-benzimidazole) (TPBi, ~45 nm)/lithium fluoride (LiF, 1.5 nm)/Al (50 nm). Elemental mapping using energy-dispersive X-ray (EDX) spectroscopy (Fig. 4b and Supplementary Fig. S14) shows uniformly distributed Cu and I atoms throughout the film, consistent with the uniformly distributed nanoclusters. The LED shows a broad EL spectrum centered at 557 nm with a FWHM of ~120 nm (Fig. 4d), which has a Commission Internationale de l’Eclairage (CIE) color coordinate of (0.43, 0.53) (Supplementary Fig. S15), a CCT of around 3840 K, and a CRI of ~45. The EL spectra remain unchanged at different bias voltages, exhibiting excellent color stability. Compared with the spectrum of the traditional white LEDs, the EL spectra of our device omit intense blue light components (Supplementary Fig. S16). The angular-dependent EL intensity of the LED follows the Lambertian profile (Supplementary Fig. S17). The device turns on at 3.9 V (corresponding to a luminance of 1 cd m^−2^) due to the relatively large bandgap of the nanocluster (Fig. 4e). Thanks to the efficient carrier injection and transport enabled by the optimized hole-transporting layers and the conductive 35DCzPPy molecules, the champion device shows a maximum luminance of up to 49,242 cd m^−2^, the record value among cluster LEDs to the best of our knowledge (Fig. 4e). A peak EQE and LER of 13.05% and 27.5 lm W^−1^ are achieved (Fig. 4f), respectively, representing state-of-the-art broadband LEDs based on a single emitter (Supplementary Tab. S4). Owing to the dual-mode emission of the nanoclusters to inhibit exciton accumulation and quenching, the device shows decent EQE roll-off from 1000 to 10,000 cd m^−2^. Moreover, the one-step solution deposition endows the great reproducibility of the LEDs, with an average EQE of ~11.7 ± 0.5% for 81 devices from 9 batches (Fig. 4g). We also fabricate bright large-area LEDs with an active size of 3 × 3 cm^2^ and flexible devices through the one-step synthesis-deposition process (Fig. 4h–i). These devices exhibit high uniformity with a maximum EQE of the large-area devices up to 7.6% (Supplementary Fig. S18), showing the great universality of our method.

**Fig. 5:**
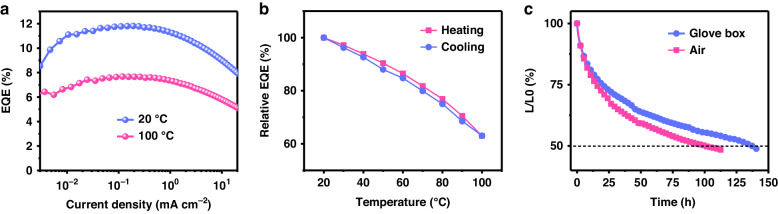
Thermal and operating stability of the LEDs. **a** EQE–current density curves of a device at 20 and 100 °C. **b** Evolution of EQE values of a device during heating and then cooling in the range of 20 to 100 °C in N_2_ under an operating current density of 1 mA cm^−2^. To ensure that the temperature of the device is consistent with the indicated one, each temperature is held for 5 min before testing. **c** Operational stability result of two unencapsulated devices in an N_2_-filled glove box and at ambient conditions (temperature, 20–25 °C; relative humidity, 45–65%), respectively. The initial luminance (*L*) is 100 cd m^−2^

We examine the thermal and operating stability of the nanocluster-based LEDs. Under operating conditions as high as 100 °C, the EQE of the device maintains above 60% of its value at room temperature (Fig. 5a). Notably, the LED efficiency is completely reversible when cooling down to 20 °C (Fig. 5b), consistent with the excellent thermal resistance of the nanocluster. The operational stability of the device is estimated in a nitrogen-filled glove box and the air without encapsulation. Remarkably, the half-lifetime (defined as the time taken for the luminance to decrease to 50% of the initial value) exhibits similar decay in the air compared with that in the nitrogen atmosphere, reaching 137 h and 13 h at an initial luminance of 100 cd m^−2^ and 1000 cd m^−2^ (Fig. 5c and Supplementary Fig. S19), respectively. These results foreshadow the promising lighting application of the CuI nanocluster-based broadband LED.

Finally, the one-step synthesis-deposition of nanocluster can be extended to other types of inorganic cores. The in-situ deposited films containing 35DCzPPy and CuBr or CuCl also show broadband emission. Without extensive optimization, the LEDs based on [35DCzPPy]_4_Cu_2_Br_2_ and [35DCzPPy]_4_Cu_2_Cl_2_ show a maximum EQE of ~2.3% and ~0.9% (Supplementary Fig. S20), respectively, with red-shifted CIE coordinates of (0.49, 0.49) and (0.54, 0.45) in sequence.

## Discussion

We develop a feasible strategy of one-step synthesis-deposition of nanocluster for fabricating ultra-broadband LEDs based on a single emitter. It is indeed encouraging to see that the in-situ solution deposited CuI nanocluster-composed film exhibits bright luminance, uniform, and homogenous characteristics, excellent ambient resistance, and thermal stability, thus enabling efficient ultra-broadband LEDs with outstanding ambient and high-temperature operation performance. The superiority in the rigidity of nanoclusters combined with the earth-rich and environmentally-friendly nature of CuI manifests the bright prospect of CuI nanoclusters to achieve broadband LED for lighting. We anticipate that the device efficiency and operational stability of the CuI nanocluster-based LEDs can be further optimized through rational ligand design.

## Materials and methods

### Materials

CuI (99.999%), CuBr (99.99%), CuCl (99.99%), tetrahydrofuran (99.9%), dichloromethane (99.9%), nafion perfluorinated resin solution (PFI; 5 wt.% in lower aliphatic alcohols and water, contains 45% water), and LiF (99.99%) were purchased from Macklin. Chlorobenzene (99.5%, anhydrous), acetonitrile (99.9%), and DBFDP (98%) were purchased from Aladdin. DMSO (99.8%, anhydrous) was purchased from Alfa Aesar. 35DCzPPy (99%), 26DCzPPy (99%), TmPyPB (99%), and TPBi (99%) were purchased from Xi’an Yuri Solar Co., Ltd. PEDOT:PSS (Clevios P VP Al 4083) was purchased from Heraeus. PF8Cz was purchased from Dongguan Volt-Amp Optoelectronics Technology Co., Ltd. Bifunctional *bis*-benzophenone (BP-BP; 97%) was purchased from Shanghai Accela ChemBio Co., Ltd.

### Preparation of the nanocluster films

The precursor solution for the [35DCzPPy]_4_Cu_2_I_2_ nanocluster film was prepared by mixing a 35DCzPPy-chlorobenzene solution with a CuI-acetonitrile solution at a volume ratio of 4 to 1. For the optimized molar ratio of 35DCzPPy to CuI (2.7 to 1) in the precursor solution, the concentrations of the 35DCzPPy solution and the CuI solution are 18 mg mL^−1^ and 9 mg mL^−1^, respectively. The CuI-acetonitrile solution was replaced by CuBr-acetonitrile or CuCl-acetonitrile solution to prepare the precursor solutions for the [35DCzPPy]_4_Cu_2_Br_2_ or [35DCzPPy]_4_Cu_2_Cl_2_ nanocluster films, respectively. For the 26DCzPPy, TmPyPB, and DBFDP chelated nanocluster films, the precursor solutions were prepared by mixing the corresponding ligand-chlorobenzene solutions with CuI-acetonitrile solution. For the spin-coating method, the nanocluster films were deposited by spin-coating the precursor solution at 2000 r.p.m for 60 s. For the blade coating method, the precursor solution was blade-coated onto the substrates with a gap of 500 μm at a movement speed of 30 mm s^−1^.

### Optical characterizations

The absolute PL QYs of the nanocluster films were measured through an integrating sphere integrated with a fluorescence spectrophotometer (FLS 1000, Edinburgh). The PL spectra and PL decay of the nanocluster films were recorded using a steady/transient fluorescence spectroscopy system (OmniFluo 900, Zolix). A xenon lamp and a 266 nm pulsed laser diode (FQSS266-Q2, CryLas) were used as excitation sources for the steady PL spectra and PL decay characteristics of the nanoclusters, respectively. The temperature-dependent PL decay spectra were measured with a temperature controller over a range of 77–300 K. The absorption spectra of the nanocluster films were measured with an ultraviolet−visible−near-infrared spectrophotometer (Cary 5000, Agilent).

### Evaluation of TADF and PH proportions

PL decay curves at different temperatures were fitted to calculate the average lifetimes. Then the lifetime-temperature curve was fitted according to Eq. 1 to calculate *ΔE*_ST_, *τ*(S_1_), and *τ*(T_1_). The TADF and PH proportions in the total emission at each temperature can be evaluated as^29^:

2 $${I}_{{\rm{tot}}}={I}_{{\rm{TADF}}}+{I}_{{\rm{PH}}}$$

3 $$\frac{{I}_{{\rm{PH}}}}{{I}_{{\rm{tot}}}}=\frac{1}{1+\frac{\tau ({{\rm{T}}}_{1})}{3\cdot \tau ({{\rm{S}}}_{1})}\exp \left(-\frac{\varDelta {E}_{\text{ST}}}{{k}_{\text{B}}T}\right)}$$

in which *I* refers to emission intensity, and the subscripts of tot stands for total emission.

### DFT calculations

The geometry optimization is conducted based on DFT theory via the Gaussian 16 program, and the structural parameters of the molecule (bond length and angle) are obtained. The Bhandhlyp functional is combined with 6-31g* and sdd basis set. The all-electron basis set 6-31g* is applied for C, H, and N elements. The Stuttgart pseudopotential basis set is applied for the I and Cu elements. The excited state properties of the molecule are computed by TDDFT theory based on the optimized structure. The functional is changed to TPSSh with the same basis set, and the Multiwfn program^40^ is introduced for the molecular NTO analysis^41^.

### EXAFS sample preparation

The powder sample was synthesized by a one-step method. 35DCzPPy (210 mg) and CuI (30 mg) were put into 10 mL chlorobenzene and stirred for 24 h at 80 °C to form a yellow-green suspension, then centrifuged at 9000 r.p.m. for 3 min to collect the precipitate. It was further washed three times by chlorobenzene and then dried at 50 °C in a N_2_-filled glove box.

### EXAFS data analysis

Data reduction, data analysis, and EXAFS fitting were performed and analyzed with the Athena and Artemis programs of the Demeter data analysis packages that utilize the FEFF6 program^42^ to fit the EXAFS data. The energy calibration of the sample was conducted through a standard Cu foil, which as a reference was simultaneously measured. A linear function was subtracted from the pre-edge region, then the edge jump was normalized using Athena software. The *χ*(*k*) data were isolated by subtracting a smooth, third-order polynomial approximating the absorption background of an isolated atom. The *k3*-weighted *χ(k)* data were Fourier transformed after applying a Hanning window function (*Δk* = 1.0). For EXAFS modeling, the global amplitude EXAFS (*CN, R, σ*^2^, and *ΔE*_0_) were obtained by nonlinear fitting, with least-squares refinement, of the EXAFS equation to the Fourier-transformed data in *R*-space, using Artemis software, EXAFS of the Cu foil is fitted and the obtained amplitude reduction factor *S*_0_^2^ value (0.900) was set in the EXAFS analysis to determine the coordination numbers (*CNs*) in the Cu–N, Cu–Cu and Cu–I scattering path in the sample.

### LED fabrication

The fabrication processes of LED devices are shown in Supplementary Fig. S21. Patterned ITO glass substrates were first cleaned by ultrasonication with acetone, deionized water, and ethanol, and then treated with oxygen plasma for 10 min before use. A mixed solution of PEDOT:PSS and PFI (at a volume ratio of 1:1) was spin-cast at 500 r.p.m. for 10 s, then 4000 r.p.m. for 45 s, followed by annealing on a hot plate at 150 °C for 15 min in air conditions. The substrates were cooled and transferred into an N_2_-filled glove box. PF8Cz solution (in chlorobenzene; 8 mg mL^−1^; with 0.8 mg mL^−1^ BP-BP crosslinker added) was spin-coated at 2000 r.p.m. for 45 s, and baked at 120 °C for 60 min and simultaneously irradiated with UV light (λ = 365 nm) to form the crosslinked HTLs. The nanocluster films were fabricated thereon by spin-coating as described above, followed by annealing on a hot plate at 150 °C for 5 min to remove the remaining solvent. Finally, the substrates were transferred into a high-vacuum thermal evaporator, where TPBi, LiF, and Al were deposited layer by layer through a shadow mask at a pressure below 5 × 10^−4^ Pa. The active sizes of the small-area and large-area devices are 4 mm^2^ and 9 cm^2^, respectively.

### LED characterization

The current density–voltage–luminance characteristics and EL spectra were measured with a custom-built system^43^ consisting of a digital source meter (Keithley 2400) and an integrating sphere (FOIS, Ocean Optics) coupled to a spectrometer (QE Pro, Ocean Optics). The operational lifetimes of the LEDs were measured using a commercial LED lifetime test system (Guangzhou Crysco Equipment). The temperature-dependent EQEs were measured using a device test box consisting of a silicon photodiode and a semiconductor temperature control system (Guangzhou Crysco Equipment). The angle dependence of the emission intensity of the LED was measured using a photodetector (PDA100A, Thorlabs) at a fixed distance of 200 mm from the LED.

### Other characterizations

AFM analyses were conducted with an atomic force microscope (Dimension Icon, Bruker). SEM images of the nanocluster films were obtained using scanning electron microscopy (Sigma HD-01-61, Zeiss). TOF-SIMS measurements were performed with a time-of-flight secondary ion mass spectrometer (TOF-SIMS V system, ION-TOF). The primary beam was 25 keV Bi^1+^ with a total current of 0.99 pA and a raster size of 100 × 100 μm^2^. Cs^+^ ions were used with 1000 eV ion energy, 43 nA pulse current on a 300 × 300 μm^2^ raster size to bombard and etch the film of negative ions. O^2+^ ions were used with 1000 eV ion energy, and 40 nA pulse current on a 300 × 300 μm^2^ raster size to bombard and etch the film of positive ions. XPS spectra were measured using a photoelectron spectrometer (EscaLab Xi+, Thermo Scientific) with a monochromatic Al Kα line (1486.6 eV) under an operating voltage of 12.5 kV. For the depth profiling XPS measurements, the sample was etched using an argon ion gun. Ultraviolet Photoelectron Spectroscopy (UPS) was performed by PHI 5000 VersaProbe III with He I source (21.22 eV) under an applied negative bias of 9.0 V. The thicknesses of the films were characterized with a step profiler (Dektak XT, Bruker). The cross-sectional sample of the LED was prepared using focused ion beam equipment (Helios G5 UX, Thermo Fisher Scientific), and high-resolution transmission electron microscopy (HRTEM) and EDX analyses of the cross-sectional sample of the LED were carried out using a Thermo Scientific TalosF200XG2.

### Supplementary information

Supplementary Figures and Tables

## Data Availability

The data that support the findings of this study are available from the corresponding authors upon reasonable request.

## References

[1] Sun CJ (2023). Perovskite light-emitting diodes toward commercial full-colour displays: progress and key technical obstacles. Light. Adv. Manuf..

[2] Zhong P, He GX, Zhang MH (2012). Optimal spectra of white light-emitting diodes using quantum dot nanophosphors. Opt. Express.

[3] Erdem T (2010). A photometric investigation of ultra-efficient LEDs with high color rendering index and high luminous efficacy employing nanocrystal quantum dot luminophores. Opt. Express.

[4] Zhang MM (2021). Molecular engineering towards efficient white-light-emitting perovskite. Nat. Commun..

[5] Gupta I (2021). Rare earth (RE) doped phosphors and their emerging applications: a review. Ceram. Int..

[6] Bispo-Jr AG (2021). Recent prospects on phosphor-converted LEDs for lighting, displays, phototherapy, and indoor farming. J. Lumin..

[7] Zhang H (2021). Rare earth-free luminescent materials for WLEDs: Recent progress and perspectives. Adv. Mater. Technol..

[8] Chen JW (2021). Perovskite white light emitting diodes: progress, challenges, and opportunities. ACS Nano.

[9] Ham W, Mueller HA, Sliney DH (1976). Retinal sensitivity to damage from short wavelength light. Nature.

[10] Wahl S (2019). The inner clock—blue light sets the human rhythm. J. Biophotonics.

[11] Luo JJ (2018). Efficient and stable emission of warm-white light from lead-free halide double perovskites. Nature.

[12] Zhang YQ (2022). Lead-free double perovskite Cs_2_AgIn_0.9_Bi_0.1_Cl_6_ quantum dots for white light-emitting diodes. Adv. Sci..

[13] Chen JW (2021). Efficient and bright white light-emitting diodes based on single-layer heterophase halide perovskites. Nat. Photonics.

[14] Chen H (2021). Efficient and bright warm-white electroluminescence from lead-free metal halides. Nat. Commun..

[15] Ma ZZ (2020). Stable yellow light-emitting devices based on ternary copper halides with broadband emissive self-trapped excitons. ACS Nano.

[16] Liu ZY (2023). Two-dimensional Cs_2_AgIn_*x*_Bi_1-*x*_Cl_6_ alloyed double perovskite nanoplatelets for solution-processed light-emitting diodes. Adv. Mater..

[17] Roccanova R (2019). Bright luminescence from nontoxic CsCu_2_X_3_ (X = Cl, Br, I). ACS Mater. Lett..

[18] Dong H (2023). Metal Halide Perovskite for next-generation optoelectronics: progresses and prospects. eLight.

[19] Chakraborty I, Pradeep T (2017). Atomically precise clusters of noble metals: Emerging link between atoms and nanoparticles. Chem. Rev..

[20] Fang Y (2017). A systematic approach to achieving high performance hybrid lighting phosphors with excellent thermal‐ and photostability. Adv. Funct. Mater..

[21] Jin RC (2016). Atomically precise colloidal metal nanoclusters and nanoparticles: Fundamentals and opportunities. Chem. Rev..

[22] Xie MC (2019). Highly efficient sky blue electroluminescence from ligand-activated copper iodide clusters: overcoming the limitations of cluster light-emitting diodes. Sci. Adv..

[23] Xie MC (2017). White electroluminescent phosphine-chelated copper iodide nanoclusters. Chem. Mater..

[24] Huang YZ (2020). Elaborate design of Ag_8_Au_10_ cluster [2]catenane phosphors for high-efficiency light-emitting devices. ACS Appl. Mater. Interfaces.

[25] Xu LJ (2016). High-efficiency solution-processed OLEDs based on cationic Ag_6_Cu heteroheptanuclear cluster complexes with aromatic acetylides. J. Mater. Chem. C.

[26] Zhang N (2022). Overcoming efficiency limitation of cluster light-emitting diodes with asymmetrically functionalized biphosphine Cu_4_I_4_ cubes. J. Am. Chem. Soc..

[27] Zhu K (2021). A new type of hybrid copper iodide as nontoxic and ultrastable LED emissive layer material. ACS Energy Lett..

[28] Liu XC (2014). Co-deposited Cu(I) complex for tri-layered yellow and white organic light-emitting diodes. Adv. Funct. Mater..

[29] Liu ZW (2011). A codeposition route to CuI-pyridine coordination complexes for organic light-emitting diodes. J. Am. Chem. Soc..

[30] Xu R (2022). Low-voltage driving copper iodide-based broadband electroluminescence. ACS Energy Lett..

[31] Cai C (2011). High-efficiency red, green and blue phosphorescent homojunction organic light-emitting diodes based on bipolar host materials. Org. Electron..

[32] Chaskar A, Chen HF, Wong KT (2011). Bipolar host materials: a chemical approach for highly efficient electrophosphorescent devices. Adv. Mater..

[33] Kyle KR (1991). Photophysical studies in solution of the tetranuclear copper(I) clusters Cu_4_I_4_L_4_ (L = pyridine or substituted pyridine). J. Am. Chem. Soc..

[34] Vitale M, Palke WE, Ford PC (1992). Origins of the double emission of the tetranuclear copper(I) cluster Cu_4_I_4_(pyridine)_4_: an ab initio study. J. Phys. Chem..

[35] Hashimoto M (2011). Highly efficient green organic light-emitting diodes containing luminescent three-coordinate copper(I) complexes. J. Am. Chem. Soc..

[36] Tsuboyama A (2007). Photophysical properties of highly luminescent copper(I) halide complexes chelated with 1,2-bis(diphenylphosphino)benzene. Inorg. Chem..

[37] Hofbeck T, Monkowius U, Yersin H (2015). Highly efficient luminescence of Cu(I) compounds: thermally activated delayed fluorescence combined with short-lived phosphorescence. J. Am. Chem. Soc..

[38] Leitl MJ (2014). Phosphorescence versus thermally activated delayed fluorescence. Controlling singlet-triplet splitting in brightly emitting and sublimable Cu(I) compounds. J. Am. Chem. Soc..

[39] Zhu ZQ (2015). Harvesting all electrogenerated excitons through metal assisted delayed fluorescent materials. Adv. Mater..

[40] Lu T, Chen FW (2012). Multiwfn: A multifunctional wavefunction analyzer. J. Comput. Chem..

[41] Martin RL (2003). Natural transition orbitals. J. Chem. Phys..

[42] Zabinsky SI (1995). Multiple-scattering calculations of X-ray-absorption spectra. Phys. Rev. B.

[43] Dai XL (2014). Solution-processed, high-performance light-emitting diodes based on quantum dots. Nature.

