# Supplementary material for: Efficient and bright broadband electroluminescence based on environment-friendly metal halide nanoclusters
Source: Light Sci Appl. 2024 Apr 7;13:82. doi: 10.1038/s41377-024-01427-z (PMC10999448; doi:10.1038/s41377-024-01427-z)
Supplement: Supplementary file 1 — Supplementary Figures and Tables [file 41377_2024_1427_MOESM1_ESM.docx]

**Supplementary Information for**

**Efficient and bright broadband electroluminescence based on environment-friendly metal halide nanoclusters**

Dingshuo Zhang^1^, Meiyi Zhu^1,2^, Yifan He^3^, Qingli Cao^1^, Yun Gao^1^, Hongjin Li^1^, Guochao Lu^1^, Qiaopeng Cui^1^, Yongmiao Shen^4^, Haiping He^1,2,3^, Xingliang Dai^1,2,3^* and Zhizhen Ye^1,2,3^*

^1^School of Materials Science and Engineering, State Key Laboratory of Silicon Materials, Zhejiang University, Hangzhou, 310027, China.

^2^Wenzhou Key Laboratory of Novel Optoelectronic and Nano Materials, Institute of Wenzhou, Zhejiang University, Wenzhou, 325006, China.

^3^Wenzhou XINXINTAIJING Tech. Co. Ltd., Wenzhou, 325006, China.

^4^Department of Chemistry, Zhejiang Sci-Tech University, Hangzhou, 310018, China.

*Corresponding author. Email: [shanfeng@zju.edu.cn](mailto:shanfeng@zju.edu.cn) (X. D.); [yezz@zju.edu.cn](mailto:yezz@zju.edu.cn) (Z. Y.)

**This file includes:**

Supplementary Figs. S1 to S21

Supplementary Tabs. S1 to S4

References


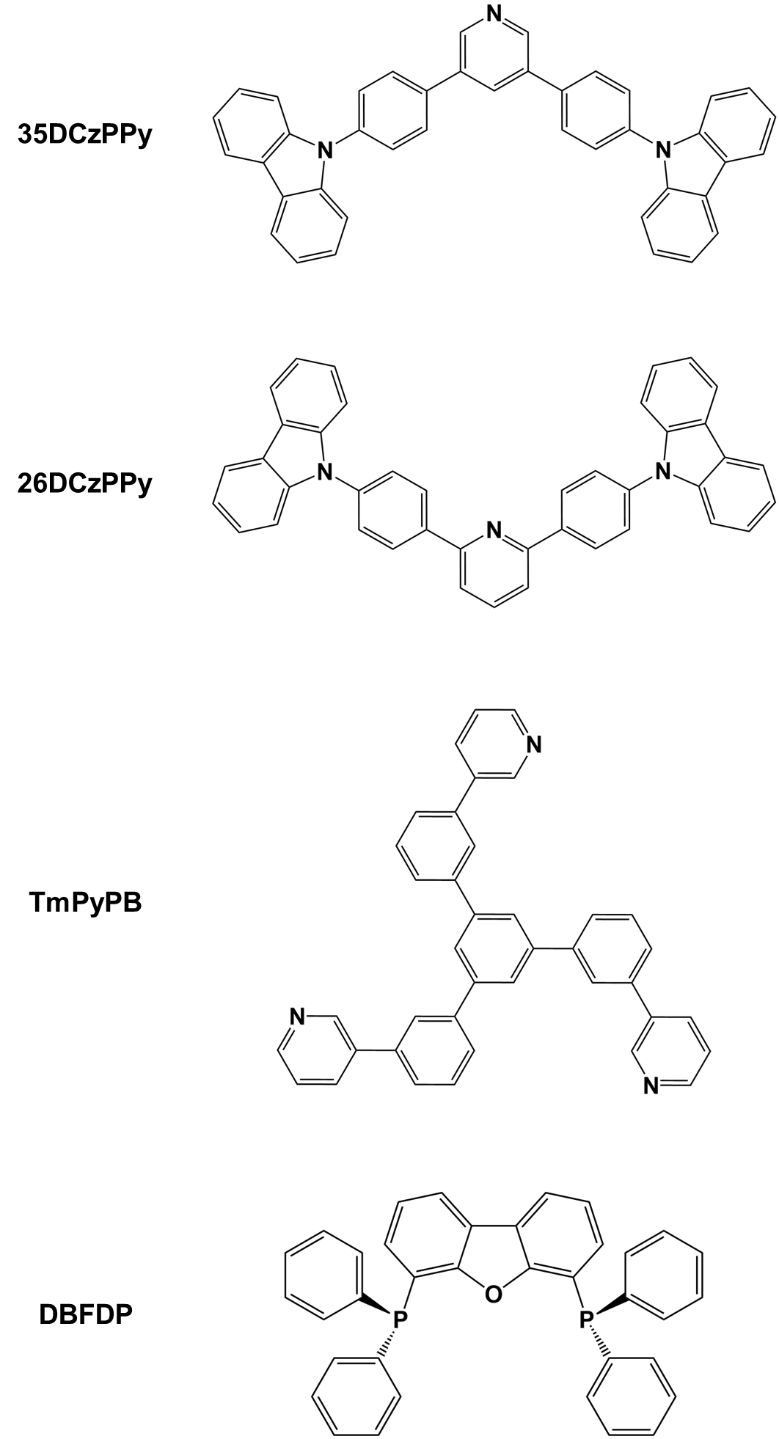


Supplementary Fig. S1 Molecular structures of 35DCzPPy, 26DCzPPy, TmPyPB, and DBFDP.


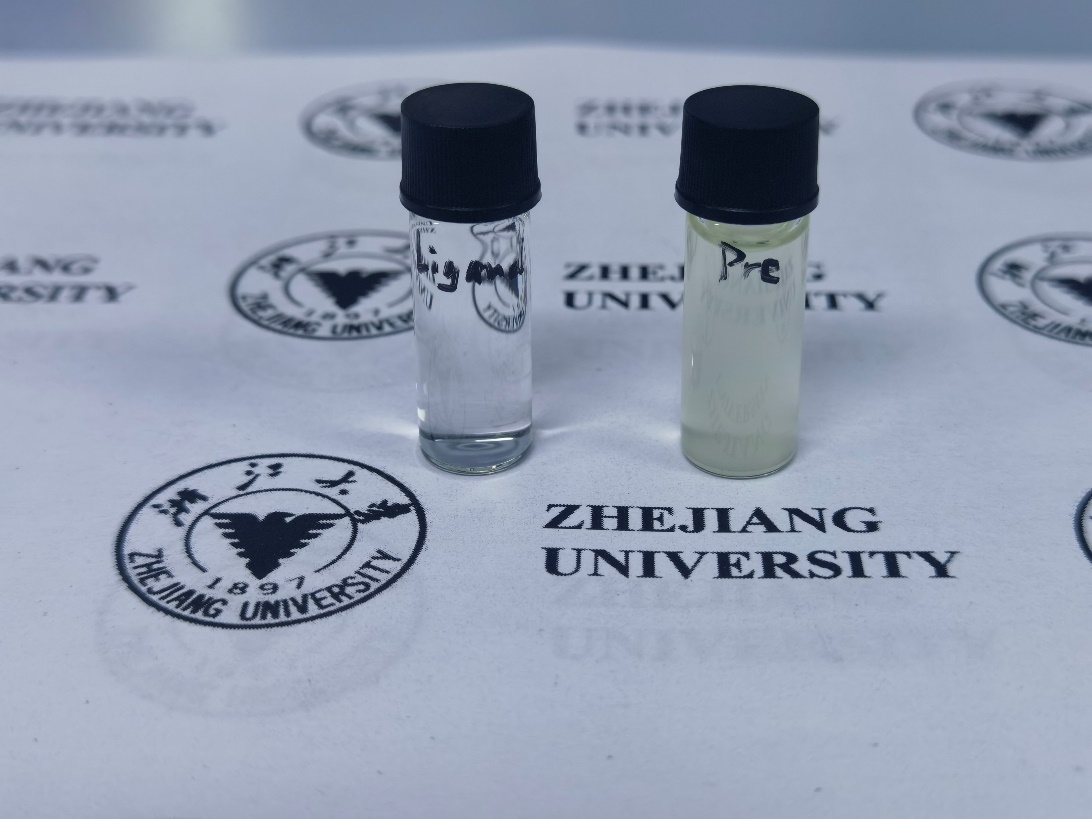


Supplementary Fig. S2 A digital photo of the dimethyl sulfoxide solution of 35DCzPPy (left), and the corresponding precursor solution prepared by mixing with the dimethyl sulfoxide solution of CuI (right). The former is clear, while the latter is cloudy due to the rapid formation and precipitation of the nanoclusters. Using a solvent that can dissolve both ligand and CuI to prepare the precursor solution is not feasible.


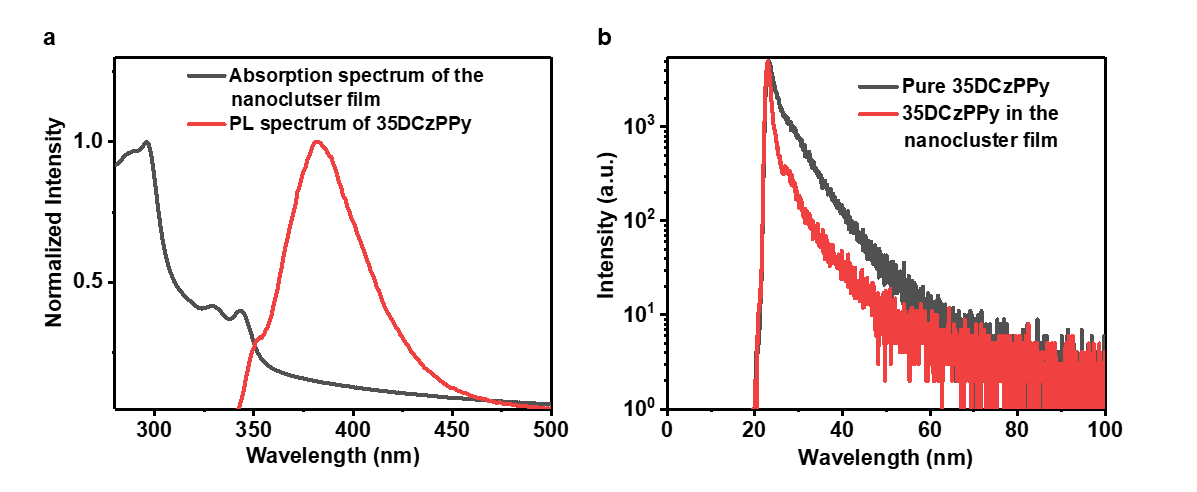


Supplementary Fig. S3 Evidence of the host-guest luminescence mechanism. a PL spectrum of 35DCzPPy and absorption spectrum of the nanocluster-composed film. The two spectra show an overlapping region at ~340 to 400 nm, which satisfies the condition of Föster energy transfer; b PL lifetime of 35DCzPPy at 380 nm in a pure 35DCzPPy film and a nanocluster film fabricated by the one-step synthesis-deposition method.


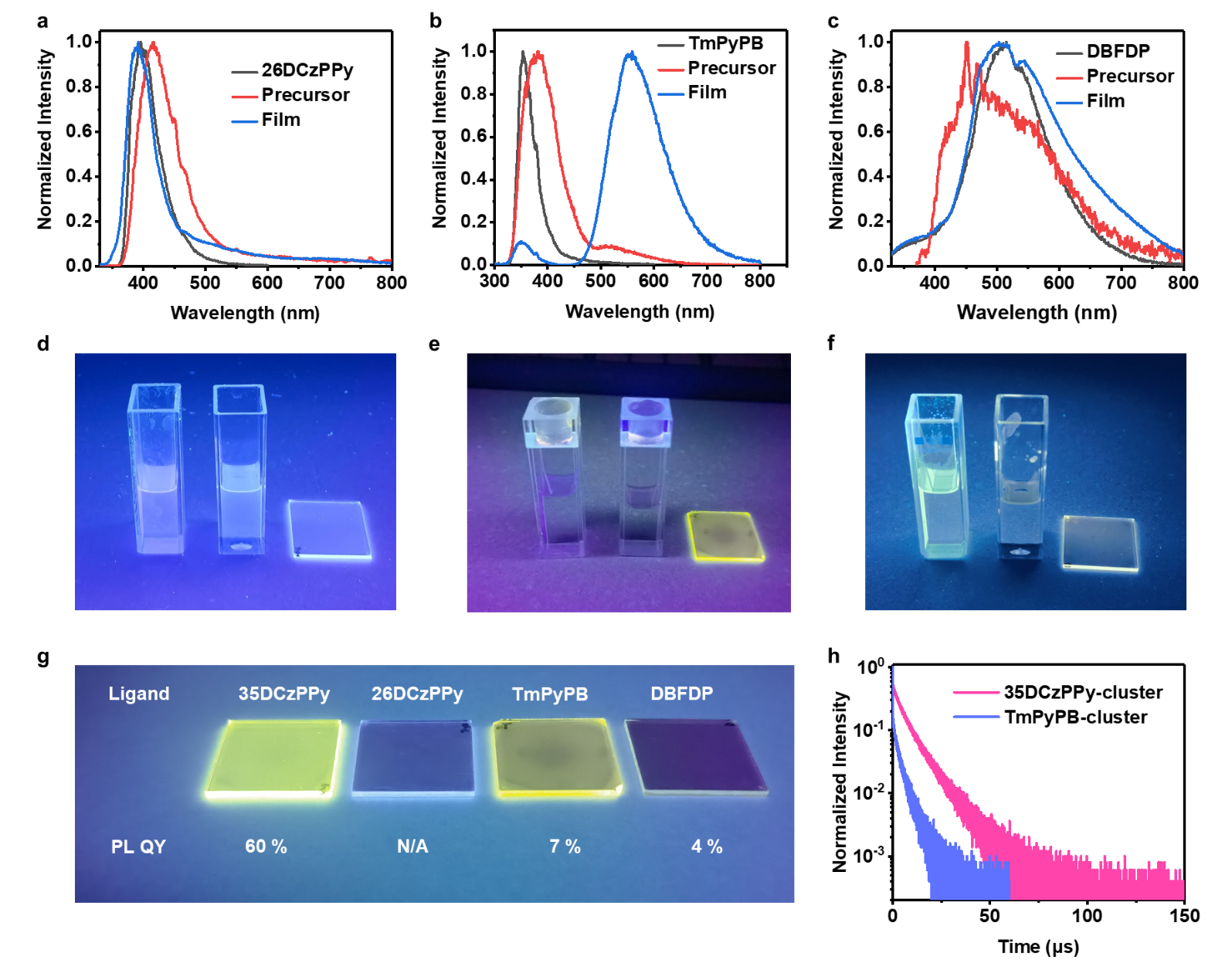


Supplementary Fig. S4 Photoluminescent properties of different ligands and corresponding precursor solutions and nanoclusters. a-f Normalized steady-state PL spectra of the ligand solution, precursor solution, and the as-prepared film as well as their digital photos in the case of 26DCzPPy (a and d), TmPyPB (b and e), and DBFDP (c and f), respectively. The excitation wavelengths are all 260 nm; g A digital photo of the nanocluster films based on different ligands under a 254 nm UV lamp, with their PL QYs labelled. Because 26DCzPPy cannot effectively coordinate with CuI to form a nanocluster with broadband emission, the PL QY is not measured; h Normalized time-resolved PL decay curves of 35DCzPPy or TmPyPB chelated nanocluster-composed films.


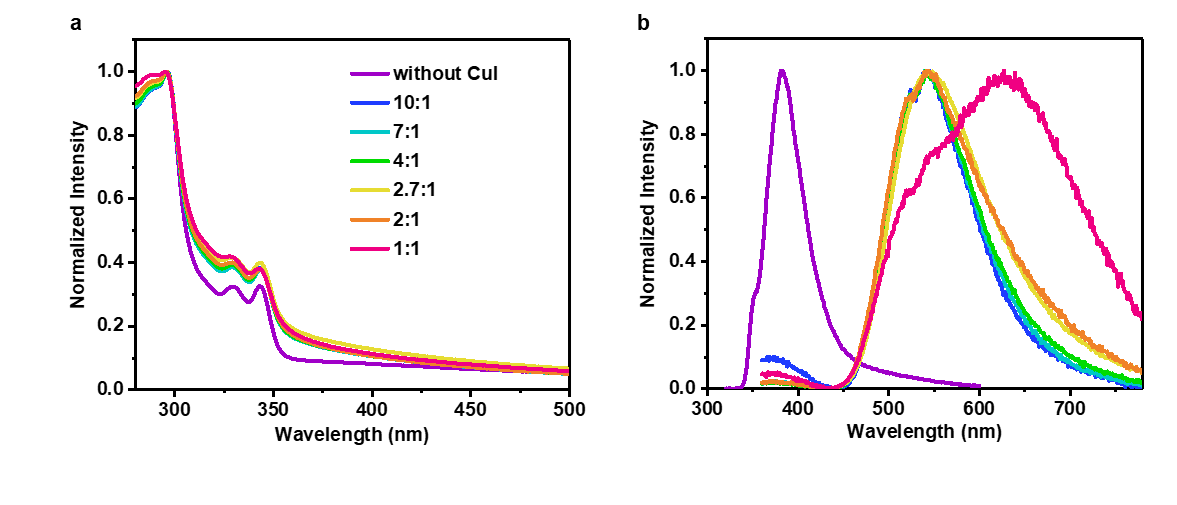


**Supplementary Fig. S5 The photophysical properties of nanocluster-composed films with different molar ratios of ligand to CuI. a**,**b** Normalized UV−vis absorption spectra (**a**) and steady-state PL spectra (**b**).


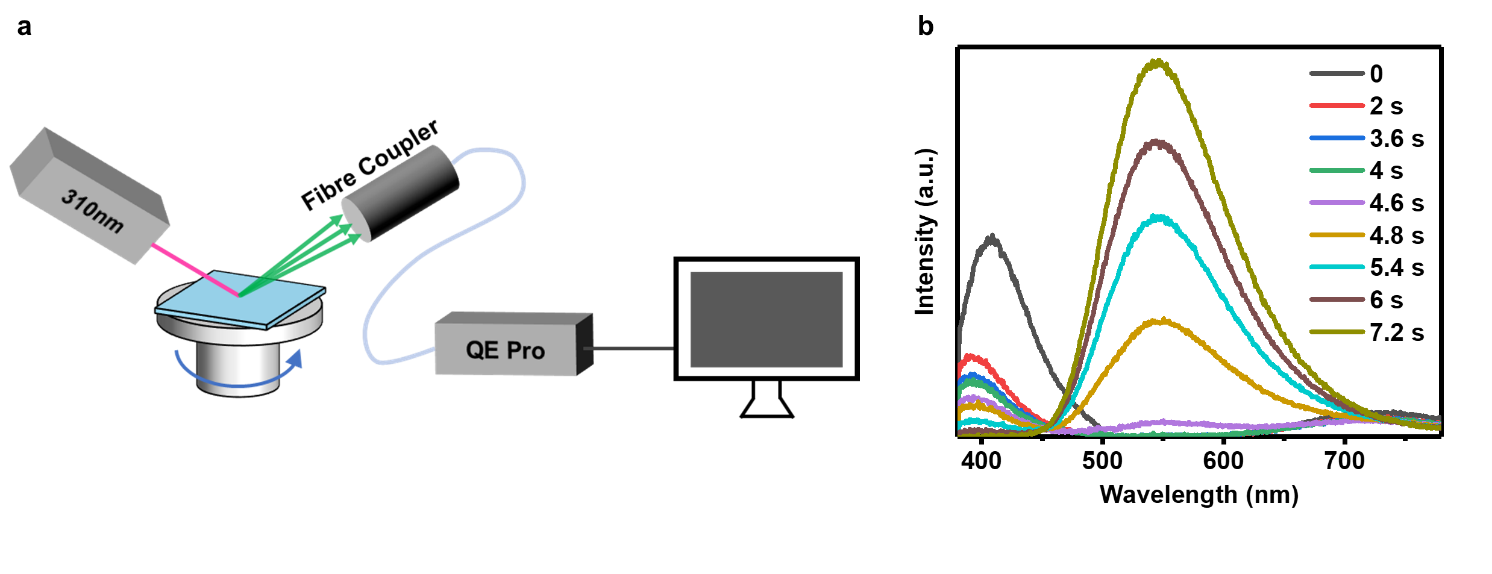


**Supplementary Fig. S6 In-situ PL measurement. a** Schematic diagram of the in-situ PL monitoring during the spin-coating process; **b** PL spectra at different times after the beginning of spin-coating.


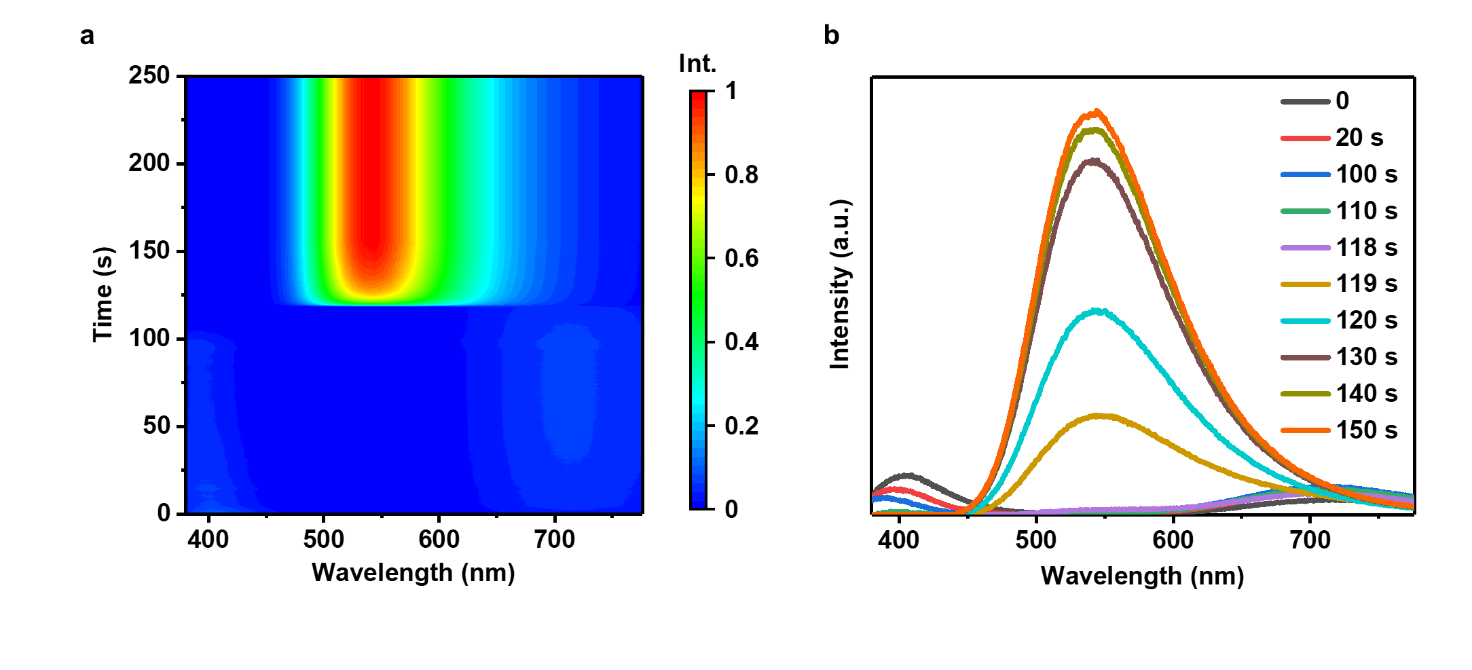


**Supplementary Fig. S7 In-situ PL measurement during the evaporation of the precursor solution under heating. a** Evolution of the PL spectra; **b** PL spectra at different times after the beginning of heating. The evolution of the PL spectra is consistent with that during the spin-coating process, which indicates that the formation of nanoclusters is caused by supersaturation induced by solvent volatilization.


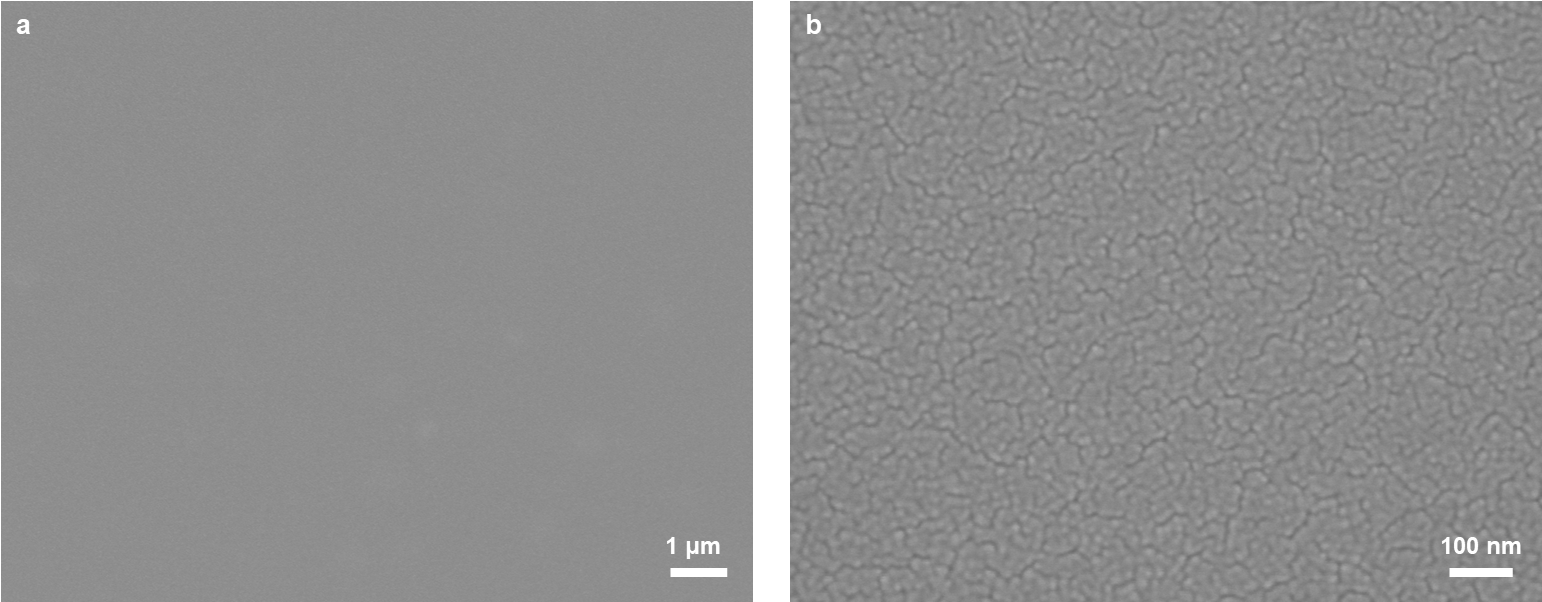


**Supplementary Fig. S8 SEM images of the nanocluster-composed film.** Scale bar: **a** 1μm; **b** 100 nm.

**
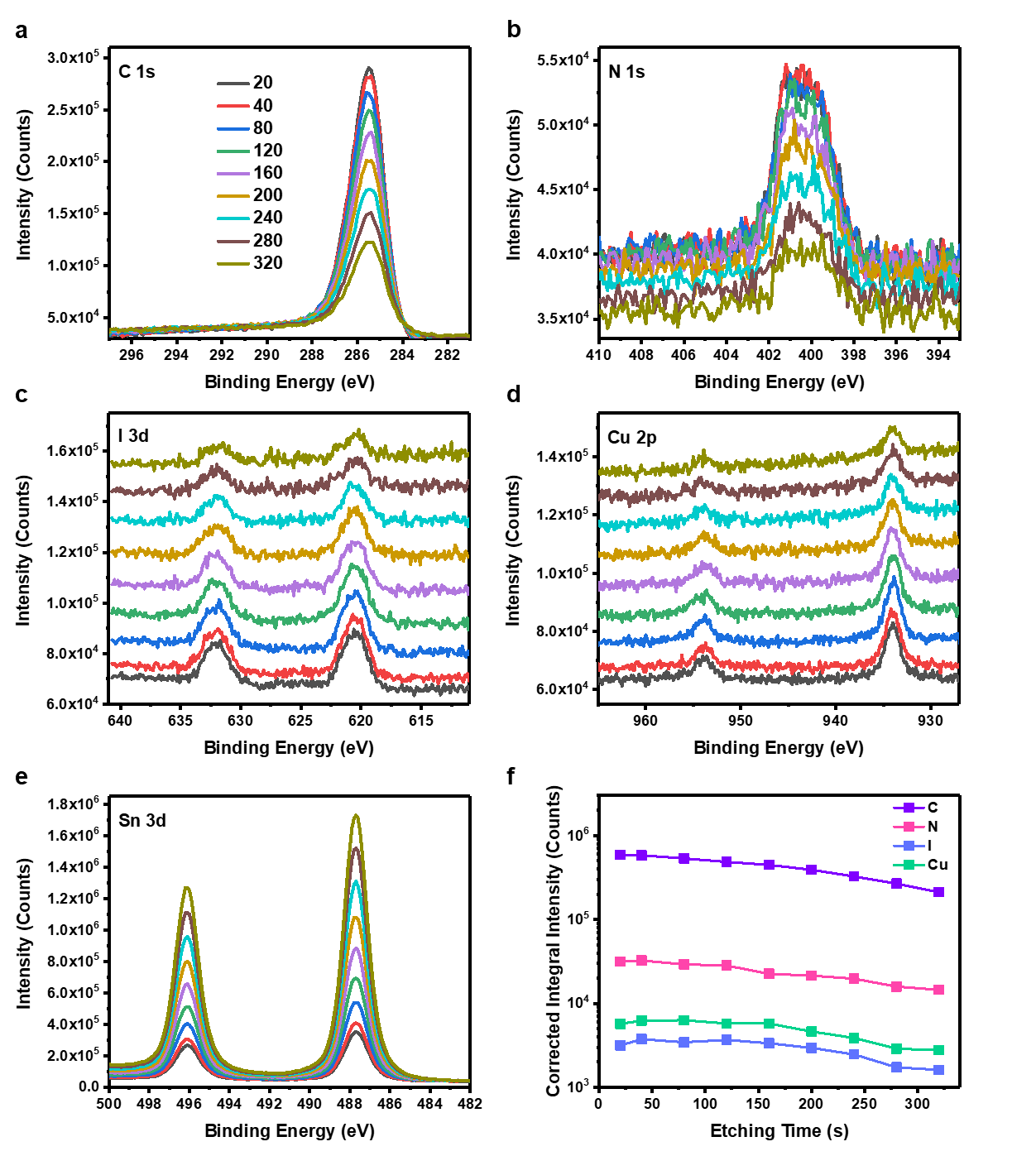
**

**Supplementary Fig. S9 XPS high-resolution spectra of the nanocluster film at different etching times. a**-**f** C 1s (**a**), N 1s (**b**), I 3d (**c**), Cu 2p (**d**), Sn 3d (**e**), and corrected integral intensity curves (**f**). Due to surface adsorption, the data are collected from the 20s after the beginning of etching. With the increase of etching time, the intensity of C, N, I, and Cu decreases gradually, while the strength of Sn increases rapidly, indicating that the nanocluster film fabricated on FTO is etched to the bottom. The corrected integral intensity is obtained by first subtracting the background baseline, and then integrating the characteristic peaks of the elements, followed by correcting with the instrument sensitivity factor for the corresponding element. The elemental atomic percentage shown in Fig. 2D is calculated based on the proportion of the corrected integrated intensity of each element to the total corrected integrated intensity.


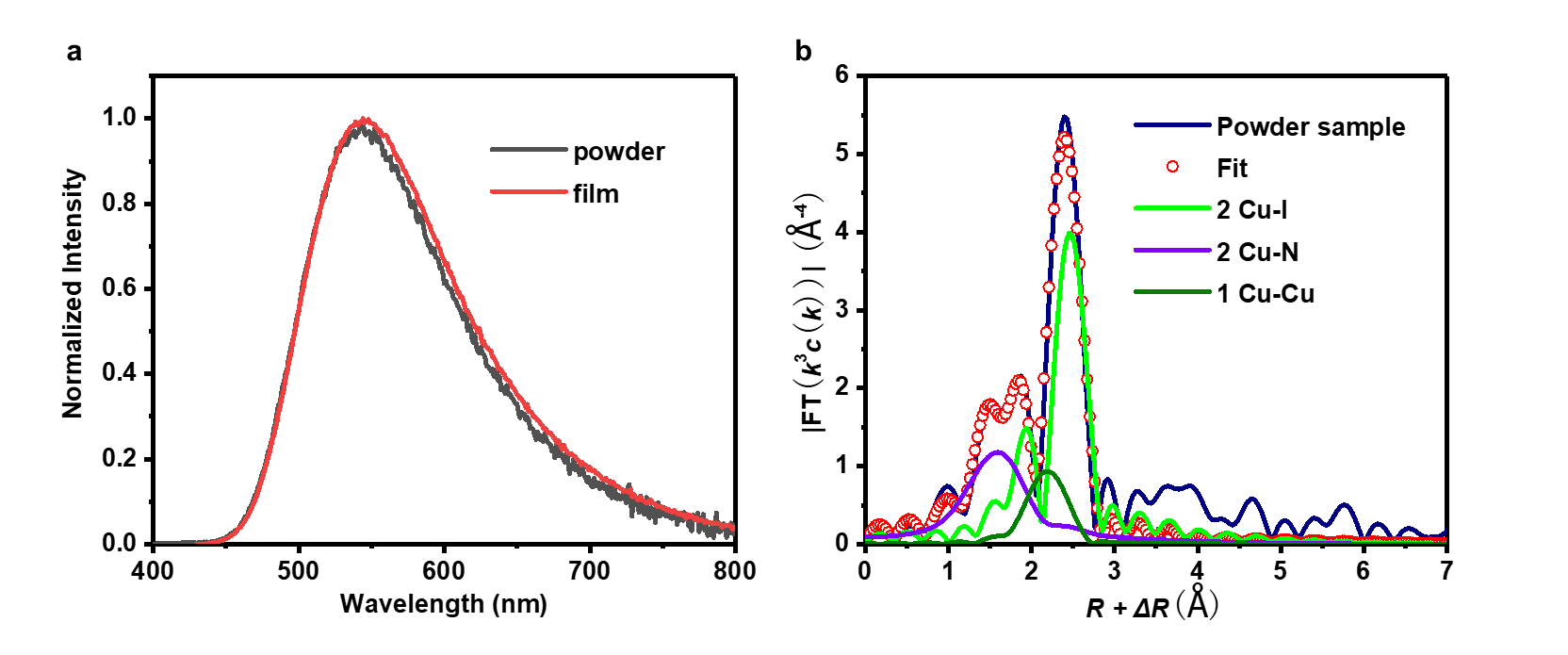


**Supplementary Fig. S10 Verification of the structure of [35DCzPPy]_4_Cu_2_I_2_ by EXAFS. a** PL spectra of the nanocluster powder and the nanocluster film (see Methods for the preparation of the powder sample). The two spectra are almost identical, indicating that the powder sample shares the same molecular structure with the film sample; **b** Non-phase-shift-corrected Fourier transform (FT) spectrum of the powder sample and the correlated fitting data. *R*, the interatomic distance in radial distribution function, in which the absorbing atom is located at the origin; *ΔR*, the phase shift. The combination of one Cu−Cu, two Cu−I, and two Cu−N paths around the Cu(I) centre gives the best fit to the data in the *R* range below ∼3.0 Å, and the features in the data at *R* > 3.0 Å are signals from further coordination layer of copper. Thus, the emitting species in the powder sample is most likely [35DCzPPy]_4_Cu_2_I_2_, as depicted in Fig. 3a.


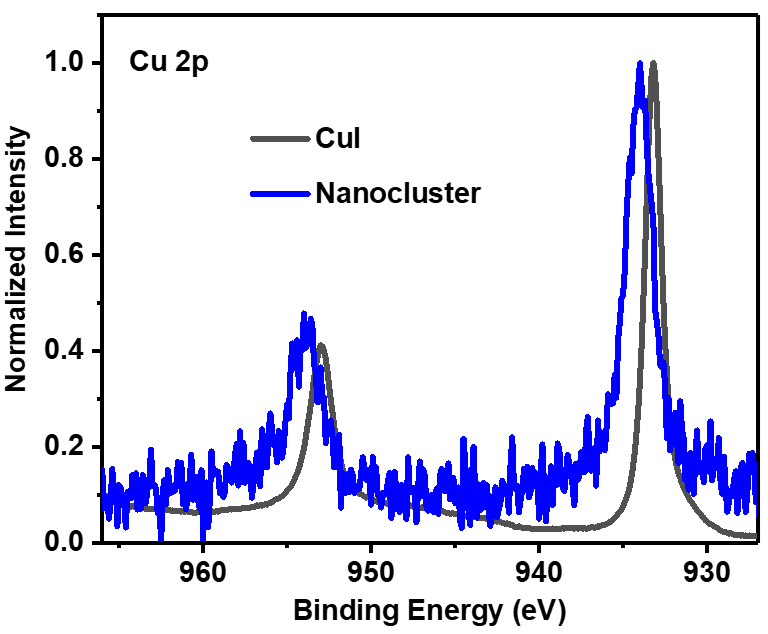


**Supplementary Fig. S11** **Normalized XPS high-resolution spectra of Cu 2p in the CuI and nanocluster films.**


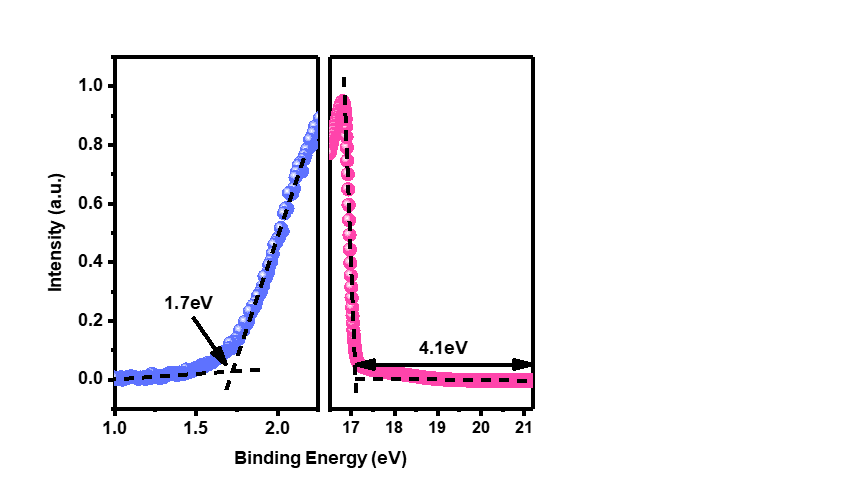


**Supplementary Fig. S12 UPS spectrum of the nanocluster film.** The Fermi level is determined to be -4.1 eV, and the gap between the HOMO and the Fermi level is 1.7eV, so the HOMO is determined to be -5.8 eV. The optical band gap obtained by the onset of the absorption spectrum is 3.5 eV, so the LUMO is determined to be -2.3 eV.


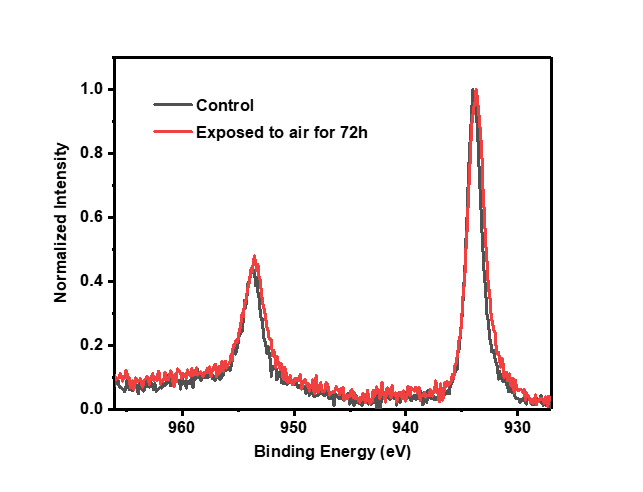


**Supplementary Fig. S13 Normalized XPS high-resolution spectra of Cu 2p in nanocluster films before or after exposure to air for 72 hours.**


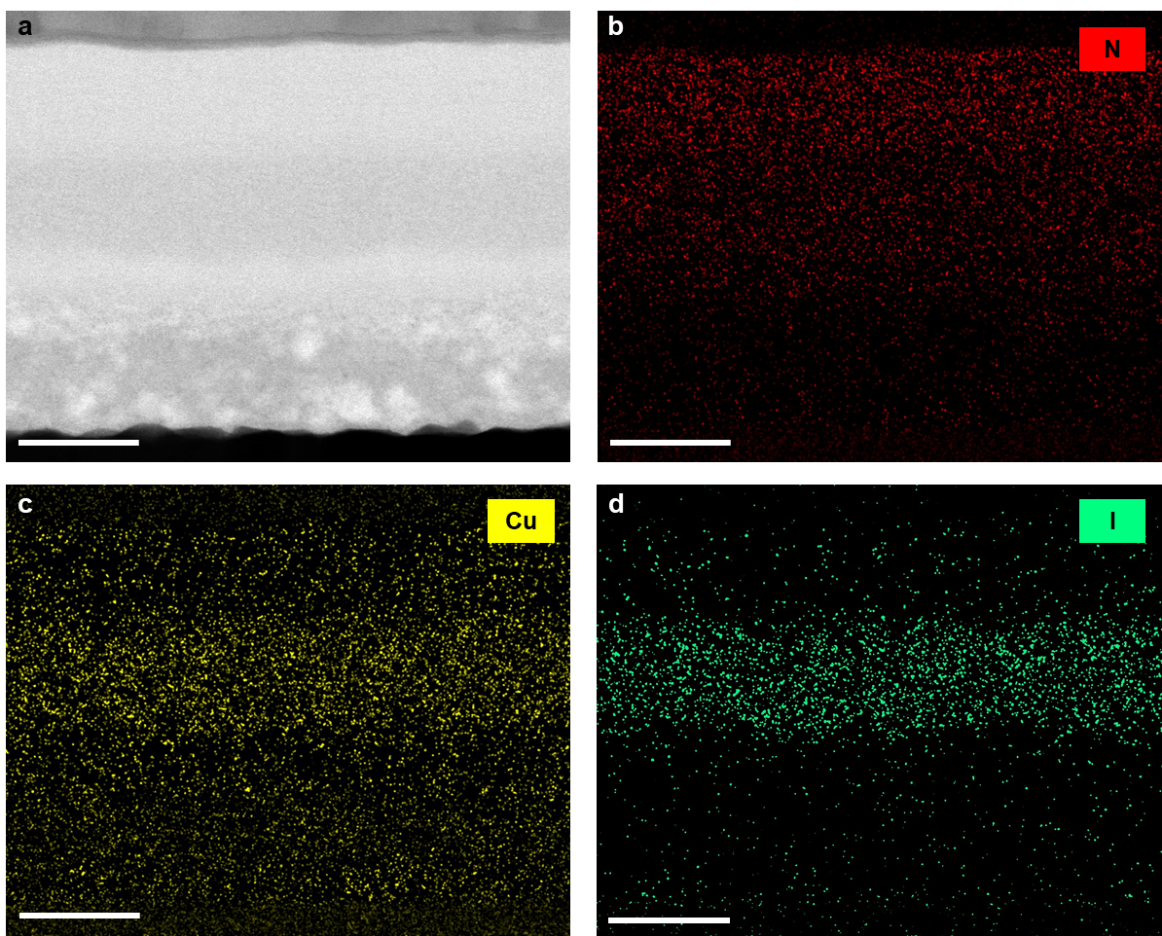


**Supplementary Fig. S14 HAADF image and the corresponding EDX images of a cross-sectional sample of the LED.** **a** HAADF image; **b-d** EDX images of N (**b**), Cu (**c**), and I (**d**). Scale bar: 50 nm.


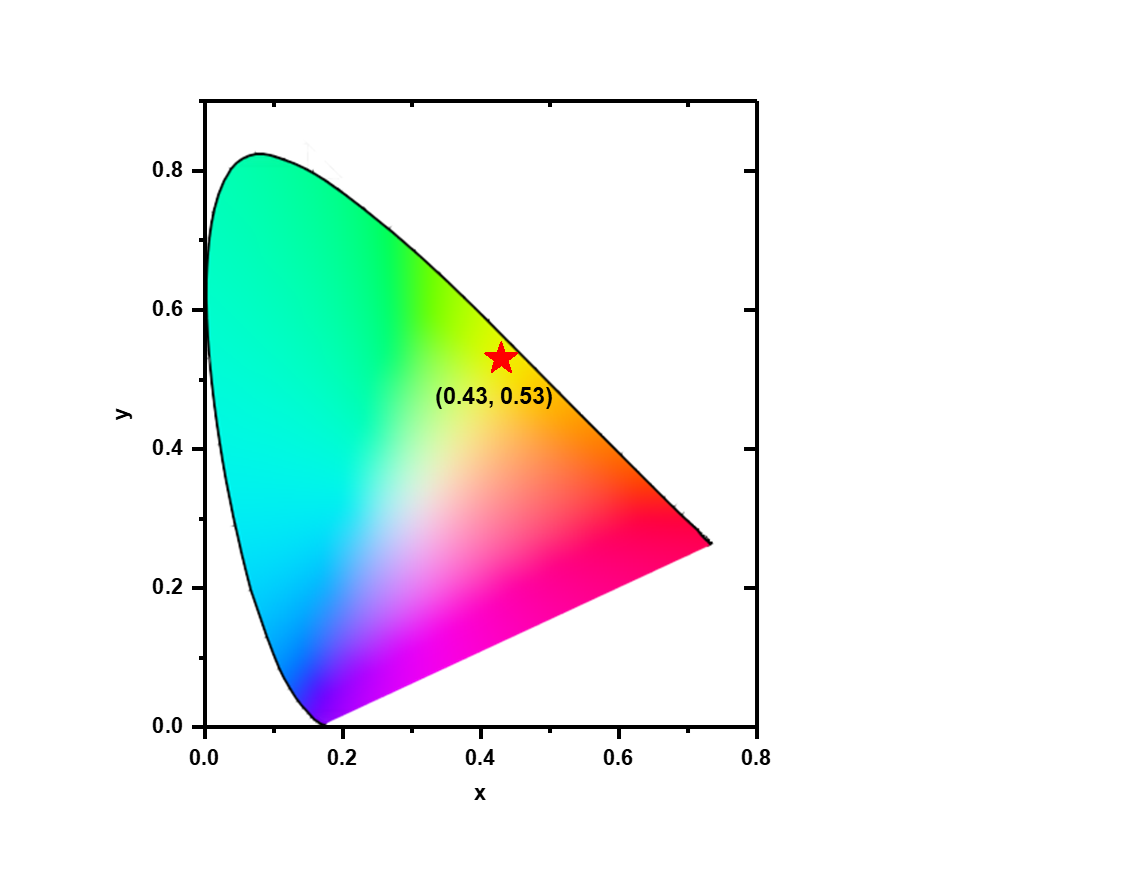


**Supplementary Fig. S15 Chromaticity diagram of the [35DCzPPy]_4_Cu_2_I_2_ nanocluster-based LEDs.**


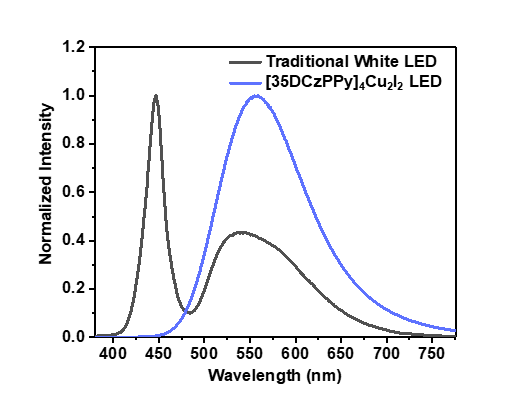


**Supplementary Fig. S16 EL spectra of the traditional white LED and the [35DCzPPy]_4_Cu_2_I_2_ nanocluster LED.**


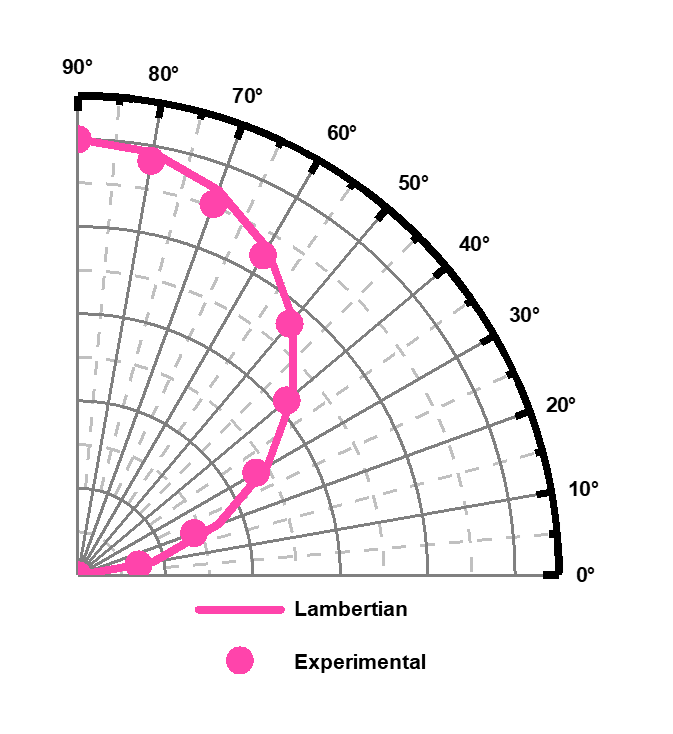


**Supplementary Fig. S17 Angular distribution of the EL intensity follows the Lambertian profile.**

**
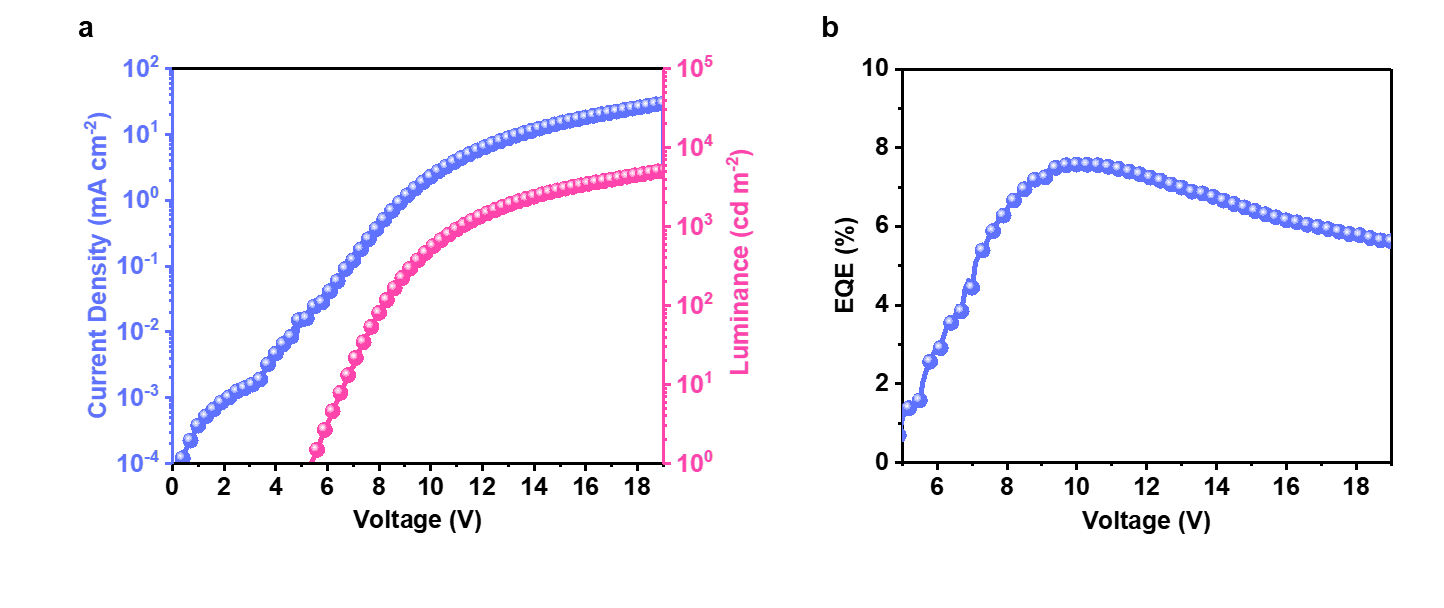
**

**Supplementary Fig. S18 Large-area LED performance. a** Current density–luminance–voltage characteristics; **b** EQE–voltage relationship of the device.

**
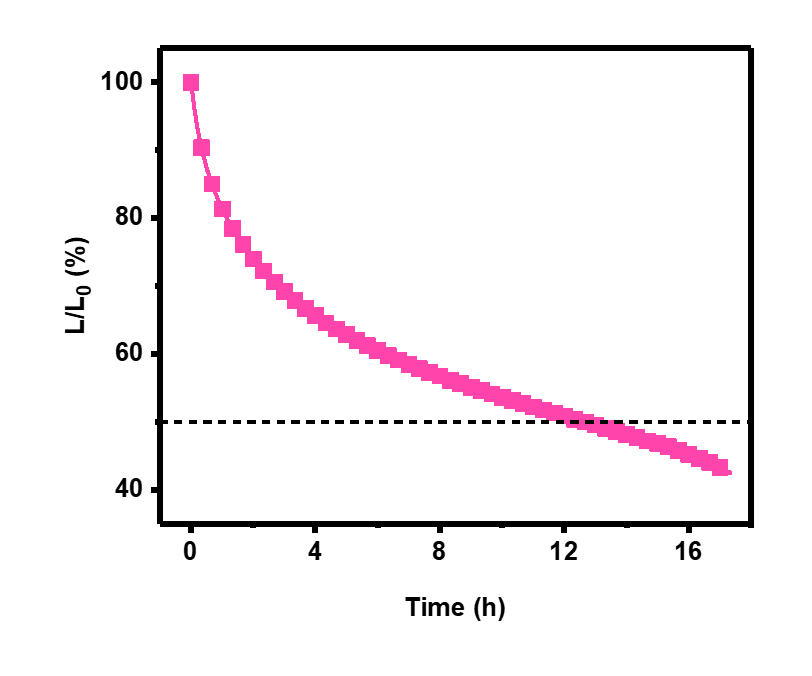
**

**Supplementary Fig. S19 Operational stability result of a device in an N_2_-filled glove box at an initial luminance (L) of 1000 cd m^-2^.**


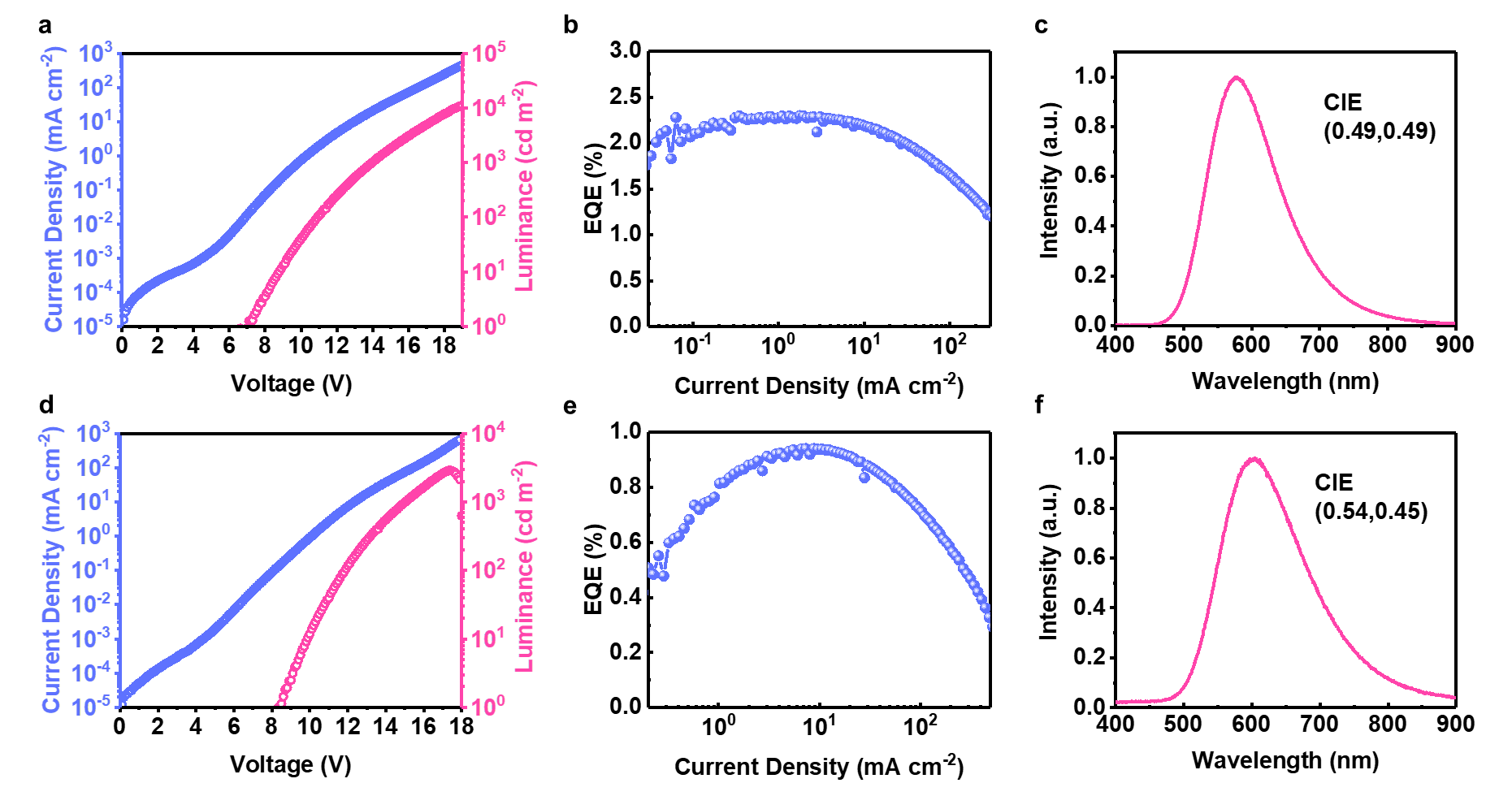


**Supplementary Fig. S20 Performances of devices based on other types of inorganic core.** **a**-**f** Current density-voltage-luminance curves, EQE-current density curves, and EL spectra of [35DCzPPy]_4_Cu_2_Br_2_ (**a**-**c**) and [35DCzPPy]_4_Cu_2_Cl_2_ (**d**-**f**).


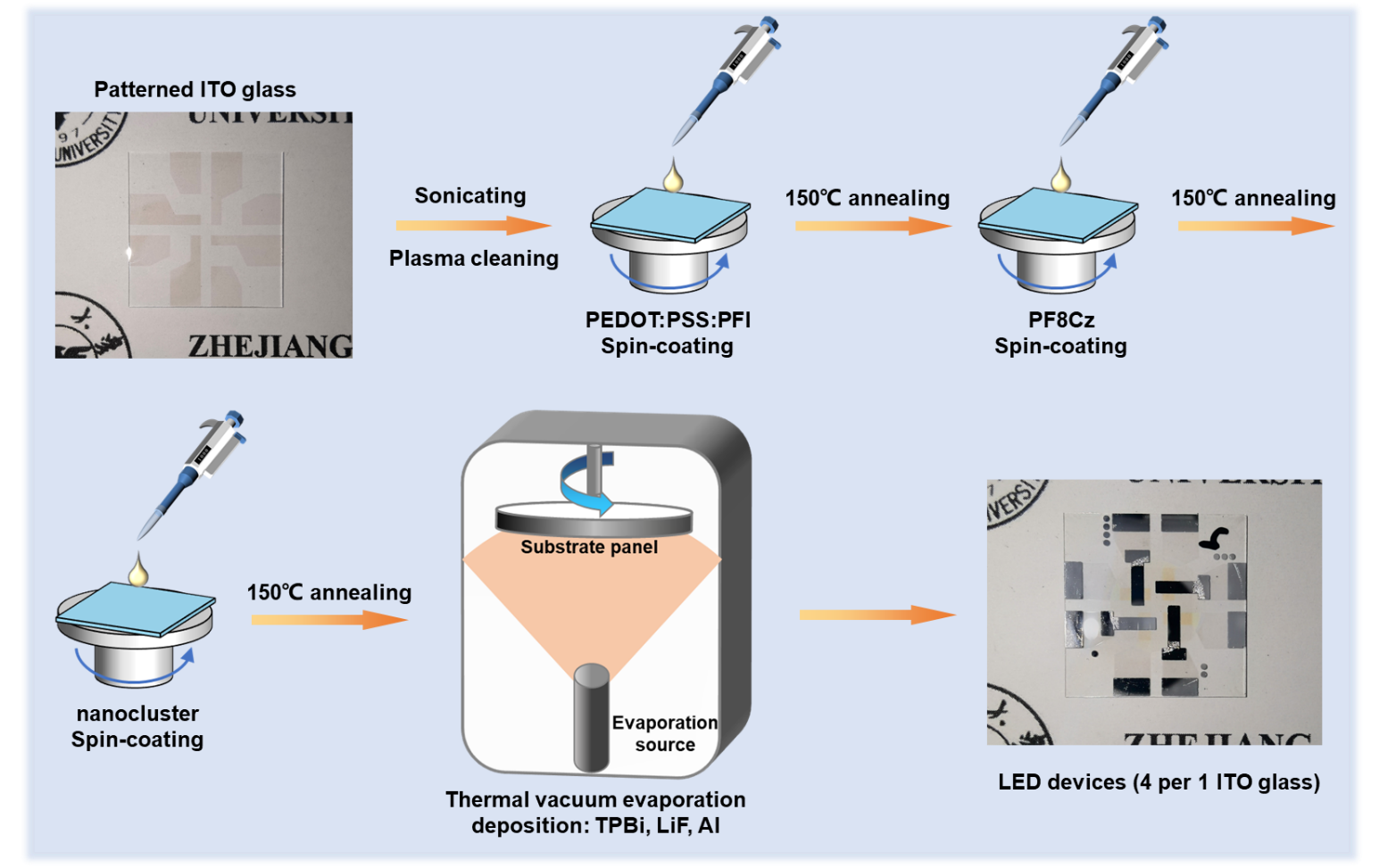


**Supplementary Fig. S21 Schematic diagram of the fabrication processes of the nanocluster LEDs.**

Supplementary Tab. S1 Solubility of 35DCzPPy, 26DCzPPy, TmPyPB, DBFDP, and CuI in several solvents at room temperature. The saturated vapor pressures of the solvents are listed to represent their volatility. The unit for solubility is expressed in mg mL^-1^.

| **Solvent**  **(saturated vapor pressure)** | **35DCzPPy** | **26DCzPPy** | **TmPyPB** | **DBFDP** | **CuI** |
| --- | --- | --- | --- | --- | --- |
| Toluene  (3.8 kPa, 25℃) | 6 | 6 | 3 | 60 | ＜0.1 |
| Chlorobenzene  (1.17 kPa, 20℃) | 18 | 18 | 8 | ＞150 | ＜0.1 |
| Dimethyl Sulfoxide  (0.049 kPa, 20℃) | 3 | 2 | 20 | 13 | 75 |
| N,N-Dimethylformamide  (0.5 kPa, 25℃) | 20 | 15 | 40 | 70 | 1.5 |
| Acetonitrile  (13.33 kPa, 27℃) | ＜0.1 | ＜0.1 | 0.4 | 2 | 33 |
| Tetrahydrofuran  (19.3 kPa, 20℃) | 50 | 30 | 15 | ＞150 | ＜0.1 |
| Dichloromethane  (46.5 kPa, 20℃) | 90 | 25 | 100 | ＞150 | ＜0.1 |
| Ethanol  (5.8 kPa, 20℃) | ＜0.1 | ＜0.1 | <0.1 | 0.7 | ＜0.1 |

Supplementary Tab. S2 EXAFS fitting parameters at the Cu *K*-edge（*Ѕ*_0_^2^=0.900）

| **Sample** | **Shell** | ***CN^a^*** | ***R*(Å)*^b^*** | ***σ*^2^(****Å^2^)*^c^*** | **Δ*E*_0_(eV)*^d^*** | ***R* factor** |
| --- | --- | --- | --- | --- | --- | --- |
| Cu foil | Cu-Cu | 12* | 2.541 | 0.0086 | 4.2 | 0.0025 |
| Powder sample | Cu-N | 2.0 | 2.092 | 0.0030 | 3.9 | 0.0159 |
|  | Cu-Cu | 1.0 | 2.491 | 0.0101 | 3.9 |  |
|  | Cu-I | 2.0 | 2.617 |  | 3.9 |  |

*S*_0_^2^ was fixed to 0.900, according to the experimental EXAFS fit of Cu foil by fixing *CN* as the known crystallographic value. *^a^CN*, coordination number; *^b^R*, the distance to the neighboring atom; *^c^σ*^2^, the Mean Square Relative Displacement (MSRD); *^d^ΔE*_0_, inner potential correction; *R* factor indicates the goodness of the fit. *This value was fixed during EXAFS fitting, based on the known structure of Cu. Fitting range: 3.0 ≤ *k* (/Å) ≤ 13.4 and 1.0 ≤ *R* (Å) ≤ 3.0 (Cu foil); 3.0 ≤ *k* (/Å) ≤ 12.0 and 1.0 ≤ *R* (Å) ≤ 3.0 (Cu sample). A reasonable range of EXAFS fitting parameters: 0.700 < *Ѕ*_0_^2^ < 1.000; *CN >* 0; *σ*^2^ > 0 Å^2^; |Δ*E*_0_| < 10 eV; *R* factor < 0.02.

Supplementary Tab. S3 Bond lengths and bond angles of the Cu_2_I_2_ core at ground state (S_0_) and excited states (S_1_ and ­T­_1_) optimized by DFT simulation.

| **Bond lengths or bond angles** | **S_0_ (Å or °)** | **S_1_ (Å or °)** | **T_1_ (Å or °)** |
| --- | --- | --- | --- |
| Bond lengths | | | |
| Cu - Cu | 2.80731 | 2.79041 | 2.80707 |
| Cu - I | 2.79465 | 2.81866 | 2.79244 |
|  | 2.77563 | 2.79766 | 2.77615 |
|  | 2.78008 | 2.78638 | 2.78115 |
|  | 2.7719 | 2.76455 | 2.77298 |
| Cu - N | 2.13127 | 2.10396 | 2.13099 |
|  | 2.11745 | 2.12108 | 2.118 |
|  | 2.13419 | 2.12609 | 2.13286 |
|  | 2.12491 | 2.13111 | 2.12558 |
| Bond angles | | | |
| N - Cu - N | 117.27232 | 117.68277 | 117.19422 |
|  | 114.41063 | 115.52109 | 114.47028 |
| N - Cu - I | 103.37881 | 105.35698 | 103.43039 |
|  | 106.47555 | 107.58456 | 106.42678 |
|  | 106.15109 | 104.9438 | 106.21899 |
|  | 105.83331 | 104.56959 | 105.76959 |
|  | 106.15528 | 106.14253 | 106.25629 |
|  | 105.84106 | 106.408 | 105.96615 |
|  | 105.65616 | 104.58218 | 105.51475 |
|  | 106.17185 | 105.17716 | 106.09298 |

(Follow the table above)

| **Bond lengths or bond angles** | **S_0_ (Å or °)** | **S_1_ (Å or °)** | **T_1_ (Å or °)** |
| --- | --- | --- | --- |
| I - Cu - I | 118.36326 | 117.27731 | 118.43513 |
|  | 118.99969 | 119.52825 | 118.93812 |
| I - Cu - Cu | 59.50636 | 59.56973 | 59.5602 |
|  | 60.02026 | 60.71921 | 59.55588 |
|  | 59.53354 | 59.30286 | 59.95834 |
|  | 59.66476 | 60.47903 | 59.66765 |
| Cu - I - Cu | 60.47338 | 59.71106 | 60.48147 |
|  | 60.8017 | 60.21812 | 60.77647 |
| N - Cu - Cu | 112.10195 | 110.71444 | 112.10951 |
|  | 130.60662 | 131.59741 | 130.68108 |
|  | 114.71017 | 110.95056 | 114.92146 |
|  | 130.87918 | 133.52699 | 130.60823 |

Supplementary Tab. S4 Comparison of our device with reported single emitter-based broadband (white or warm-white) LEDs with a FWHM of larger than 120 nm.

| **Emitter** | **EL Peak**  **(nm)** | **FWHM**  **(nm)** | **Peak EQE**  **(%)** | **Maximum Luminance**  **(cd m^-2^)** | **T_50_ @ L_0_**  **(min @ cd m^-2^)** | **Reference** |
| --- | --- | --- | --- | --- | --- | --- |
| Cs_2_Ag_0.6_Na_0.4_InCl_6_ | ~559^†^ | ~155^†^ | N/A | ~50^†^ | 10 @ ~45^†^ | ^1^ |
| heterophase α/δ-CsPbI_3_ | ~531, 690^†^ | ~178^†‡^ | 6.5 | 12200 | 230 @ ~2^†^ | ^2^ |
| CsCu_2_I_3_ | 565 | 121 | 3.1 | 1570 | N/A | ^3^ |
| CsCu_2_I_3_ | 554 | 136 | ~0.15^†^ | ~14^†^ | N/A | ^4^ |
| [DBFDP]_2_Cu_4_I_4_ | 550 | ~188^†^ | 0.73 | ~1500 | N/A | ^5^ |
| CuI complex | 560 | 123 | 4.6 | 1650 | 0.2 @ 500 | ^6^ |
| **[35DCzPPy]_4_Cu_2_I_2_** | **557** | **120** | **13.05** | **49242** | **8220 @ 100**  **780 @ 1000** | **This work** |

†These data are estimated from the figures in the corresponding references.

‡Since the EL spectrum contains more than one peak, the FWHM represents only the stronger peak in the spectrum.

**References**

1. Luo, J. et al. Efficient and stable emission of warm-white light from lead-free halide double perovskites. *Nature* **563**, 541-545 (2018).

2. Chen, J. et al. Efficient and bright white light-emitting diodes based on single-layer heterophase halide perovskites. *Nat. Photonics* **15**, 238-244 (2021).

3. Chen, H. et al. Efficient and bright warm-white electroluminescence from lead-free metal halides. *Nat. Commun.* **12**, 1421 (2021).

4. Roccanova, R. et al. Bright luminescence from nontoxic CsCu_2_X_3_ (X = Cl, Br, I). *ACS Materials Lett.* **1**, 459-465 (2019).

5. Xie, M. et al. White electroluminescent phosphine-chelated copper iodide nanoclusters. *Chem. Mater.* **29**, 6606-6610 (2017).

6. Xu, R. et al. Low-voltage driving copper iodide-based broadband electroluminescence. *ACS Energy Lett.* **7**, 4408-4416 (2022).
